# A Novel Mobile App to Identify Patients With Multimorbidity in the Emergency Setting: Development of an App and Feasibility Trial

**DOI:** 10.2196/42970

**Published:** 2023-07-13

**Authors:** Claire Barthlow Rosen, Sanford Eugene Roberts, Solomiya Syvyk, Caitlin Finn, Jason Tong, Christopher Wirtalla, Hunter Spinks, Rachel Rapaport Kelz

**Affiliations:** 1 Hospital of the University of Pennsylvania Philadelphia, PA United States

**Keywords:** clinical operationalization, delphi, development, emergency, general surgery, mHealth, mobile app, mobile app, mobile health, morbidity, multimorbidity, qualifying comorbidity set, surgery, usability

## Abstract

**Background:**

Multimorbidity is associated with an increased risk of poor surgical outcomes among older adults; however, identifying multimorbidity in the clinical setting can be a challenge.

**Objective:**

We created the Multimorbid Patient Identifier App (MMApp) to easily identify patients with multimorbidity identified by the presence of a Qualifying Comorbidity Set and tested its feasibility for use in future clinical research, validation, and eventually to guide clinical decision-making.

**Methods:**

We adapted the Qualifying Comorbidity Sets’ claims-based definition of multimorbidity for clinical use through a modified Delphi approach and developed MMApp. A total of 10 residents input 5 hypothetical emergency general surgery patient scenarios, common among older adults, into the MMApp and examined MMApp test characteristics for a total of 50 trials. For MMApp, comorbidities selected for each scenario were recorded, along with the number of comorbidities correctly chosen, incorrectly chosen, and missed for each scenario. The sensitivity and specificity of identifying a patient as multimorbid using MMApp were calculated using composite data from all scenarios. To assess model feasibility, we compared the mean task completion by scenario to that of the American College of Surgeons National Surgical Quality Improvement Program Surgical Risk Calculator (ACS-NSQIP-SRC) using paired *t* tests. Usability and satisfaction with MMApp were assessed using an 18-item questionnaire administered immediately after completing all 5 scenarios.

**Results:**

There was no significant difference in the task completion time between the MMApp and the ACS-NSQIP-SRC for scenarios A (86.3 seconds vs 74.3 seconds, *P*=.85) or C (58.4 seconds vs 68.9 seconds*,* *P=*.064), MMapp took less time for scenarios B (76.1 seconds vs 87.4 seconds, *P=*.03) and E (20.7 seconds vs 73 seconds, *P*<.001), and more time for scenario D (78.8 seconds vs 58.5 seconds, *P=*.02). The MMApp identified multimorbidity with 96.7% (29/30) sensitivity and 95% (19/20) specificity. User feedback was positive regarding MMApp’s usability, efficiency, and usefulness.

**Conclusions:**

The MMApp identified multimorbidity with high sensitivity and specificity and did not require significantly more time to complete than a commonly used web-based risk-stratification tool for most scenarios. Mean user times were well under 2 minutes. Feedback was overall positive from residents regarding the usability and usefulness of this app, even in the emergency general surgery setting. It would be feasible to use MMApp to identify patients with multimorbidity in the emergency general surgery setting for validation, research, and eventual clinical use. This type of mobile app could serve as a template for other research teams to create a tool to easily screen participants for potential enrollment.

## Introduction

In an aging United States population [1-3], most older adults have 2 or more chronic medical conditions [4], yet the majority should not be labeled as multimorbid. Multimorbidity is a specific concept that denotes an increased risk of adverse health outcomes [5,6]. Because of this and the resources required to care for people with multimorbidity, the National Institutes of Health (NIH) has developed a “multi-institute initiative to expand research on the measurement, causes, and consequences of multimorbidity” [6].

Of the various ways to define multimorbidity [5,7-9], the most straightforward is a simple count-based definition, labeling patients with 2 or more comorbidities as multimorbid [4,6,10]. However, this broad definition lacks selectivity and does not consider the interactions between comorbidities, which may confer excessive risk. In fact, the NIH highlights the importance of considering how both concordant and discordant comorbidities can interact and inform overall health [10]. In this spirit, in 2018, Silber et al [5] proposed Qualifying Comorbidity Sets as a comprehensive mechanism to account for these interactions and identify multimorbidity among surgical patients when using discharge claims data. Our previous work has found this definition of multimorbidity to offer greater selectivity and to be associated with the greater risk of poor outcomes than other count-based definitions [11,12]. Clinical operationalization of this definition, however, is limited due to (1) the difficulty in recognizing that a Qualifying Comorbidity Set may be present for an individual patient and (2) the claims-based nature of the definition.

This study offers an innovative application of Qualifying Comorbidity Sets to define multimorbidity—an adaptation of a retrospective, claims-based research tool for future use in prospective clinical investigation and eventual clinical practice. Our research team sought to develop a novel mobile app, the Multimorbid Patient Identifier App (MMApp), to easily identify patients as multimorbid in real time in the clinical setting to permit informed conversations about health status and also to enable the prospective enrollment of patients with multimorbidity in clinical trials. We hypothesize that use of MMapp will be feasible even in the emergency general surgery (EGS) setting when time is limited and stakes are high. Furthermore, this app-based strategy to efficiently identify specific patient populations for enrollment in scientific investigations has broad appeal.

## Methods

### App Development

#### Study Conception

This was a first-stage feasibility trial for a novel app called MMApp. MMApp was designed to identify patients as multimorbid, defined by the presence of a Qualifying Comorbidity Set. Qualifying Comorbidity Sets are single, double, and triple combinations of acute and chronic medical conditions identified by Silber et al [5] to be associated with a 2-fold increased risk of in-hospital mortality after general surgery, and our group has found this to extend to the EGS setting [11,12]. We chose the EGS setting to test the feasibility of MMApp, as our previous retrospective investigations regarding multimorbidity have examined EGS populations [11,12]. If MMApp is feasible for use in the EGS setting, this would support prospective investigations of similar populations. Furthermore, time is limited in the emergency setting; thus, if MMApp is feasible for use here, then it would be reasonable to speculate that it is feasible for use in an elective setting as well, and further validation could be conducted. At this point, MMApp is purely investigational and not available for public use.

#### Clinical Adaptation of Claims Definition of Multimorbidity

Qualifying Comorbidity Sets were developed using Centers for Medicare and Medicaid (CMS) claims [5]. The medical conditions that comprise these sets are defined using the standard Medicare Hierarchical Condition Category (HCC) coding system (version 22) [13-26] and indicators for disability with Current Procedural Terminology (CPT) codes. See Table S1 in Multimedia Appendix 1, which shows the full list of comorbid conditions and associated International Classification of Disease Ninth/Tenth Revision Clinical Modification (ICD 9/10-CM), CPT, and Health care Common Procedure Coding System (HCPCS) codes; see Table S2 in Multimedia Appendix 1, for Qualifying Comorbidity Sets.

A total of 5 physicians (CBR, SER, CF, JT, and RRK) and 1 data analyst (CW) translated the codes to clinical terms using a modified Delphi technique [27,28]. Four physicians independently analyzed the full list of ICD 9/10-CM, CPT, and HCPCS codes used to define each comorbid condition present in any Qualifying Comorbidity Set. Each physician developed a representative clinical definition for each claims-defined comorbid condition, along with specific included and excluded diagnoses needed to differentiate each comorbid condition from any other. Subsequently, the 5 physicians (CBR, SER, CF, JT, and RRK) and the data analyst (CW) met jointly. The group discussed one claims-defined comorbid condition at a time and compared it to the proposed clinical definitions. The group also considered the relevant inclusion and exclusion diagnoses for each clinical definition before developing a consensus agreement regarding the best possible clinical definition of each claims-defined comorbid condition. Any disagreements regarding the best possible clinical corollary were discussed, and the selection of the clinical definition was determined by a unanimous vote with equal veto power for all involved. If a veto was used by any member, further discussion and revision were required to achieve a unanimous vote of agreement. There were 3 rounds of discussion over the course of 5 weeks before a final list of clinical definitions, included diagnoses, and excluded diagnoses was determined for each comorbid condition. (See Table S3 in Multimedia Appendix 1 for clinical definition, included diagnoses, excluded diagnoses, and comparable Silber- or claims-defined comorbid condition for all comorbid conditions present in any qualifying comorbidity set.)

#### App Coding and Design

**Figure 1:**
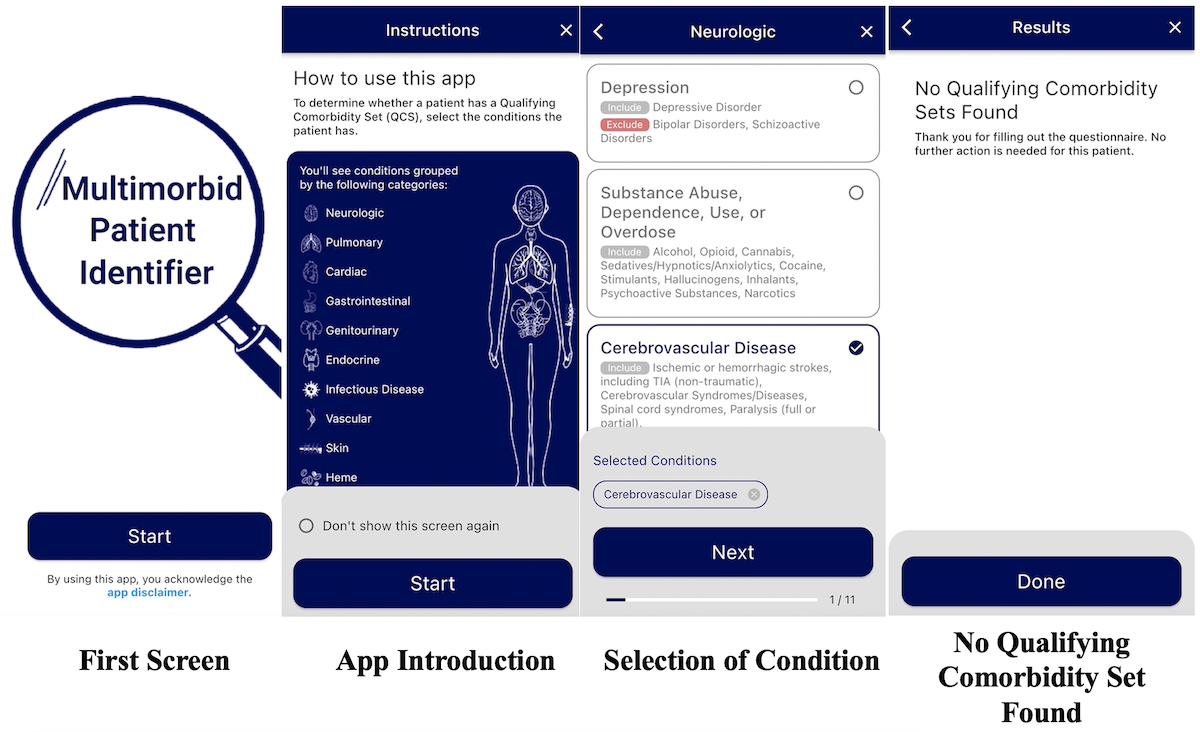
Select screenshots from Multimorbid Patient Identifier App (MMApp) for nonmultimorbid patients. Far left panel: introduction screen seen when users first open MMApp; center left panel: instruction screen for how to use the app, with an option to not see again with future use; center right panel: example of selection of a comorbid condition (Cerebrovascular Disease) within the Neurologic body system category page; far right panel: results page seen when no Qualifying Comorbidity Set is found (ie, patient is not “multimorbid”).

**Figure 2:**
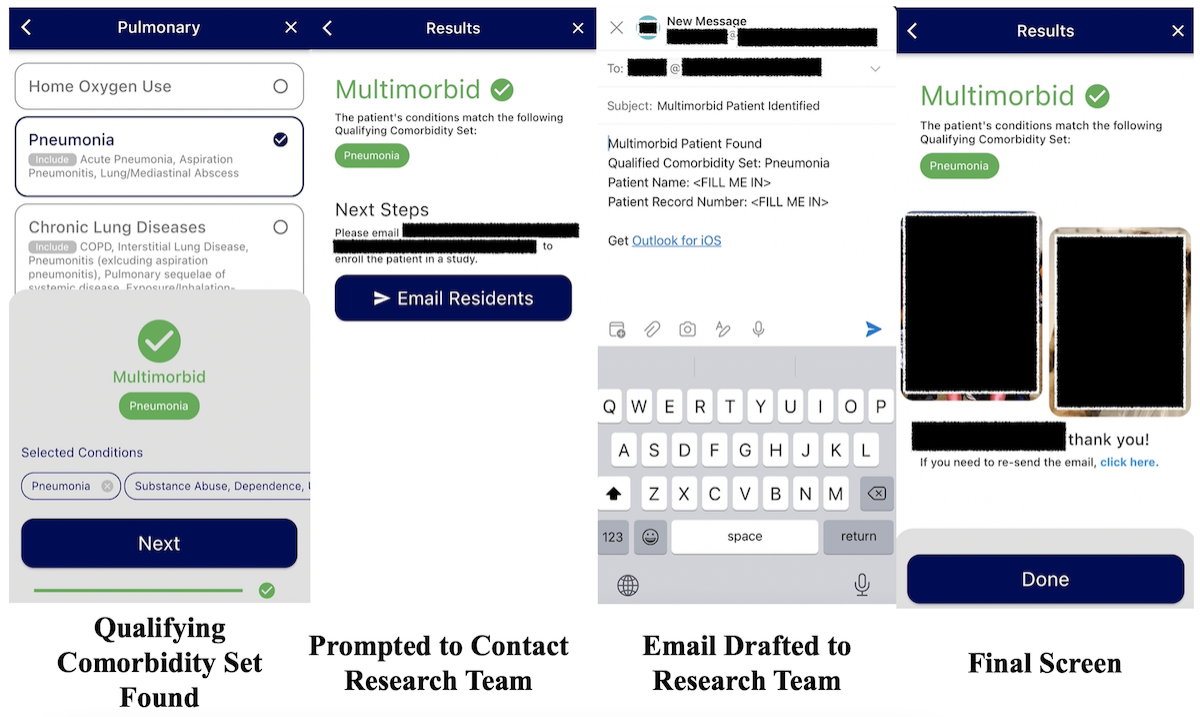
Select screenshots from Multimorbid Patient Identifier App (MMApp) for multimorbid patients. Far left panel: when the final comorbidity that makes up a Qualifying Comorbidity Set is chosen, the user is immediately notified that the patient is considered multimorbid; center left panel: results page seen when a patient is found to be multimorbid with a prompt to email study team investigators; center right panel: automatic email construction with information to email study investigators; far right panel: final results page with the opportunity to resend the email if needed.

The computer scientist on the team (HS) engineered the iOS app using the Flutter framework (Google LLC), leveraging best practices for software development such as source control management using git and GitHub (GitHub, Inc). Comorbid conditions and Qualifying Comorbidity Sets were coded into JavaScript Object Notation files included in the app bundle, which are then compared against the user’s answers to determine multimorbidity status. The app was distributed directly to study team members’ phones using Apple’s beta distribution tools, including Xcode (Apple Inc), App Store Connect, and TestFlight (Apple Inc). Graphic design (SS) and button association were created using Figma (Figma Inc; Figures 1 and 2). See Figures 1 and 2 for examples of graphic design within MMapp. (See also Table S4 in Multimedia Appendix 1 for progression through MMApp for a patient with multimorbidity, scenario D; see Table S4 in Multimedia Appendix 1 for progression through MMapp for a patient with multimorbidity, scenario E.) Comorbid conditions were organized by organ system based on input from physician team members (CBR, SER, CF, JT, and RRK).

### Feasibility Testing

#### Design

A total of 5 hypothetical patient scenarios were created by the research team and written in the History and Physical note style of the study institution (see Section 3 in Multimedia Appendix 1 for patient scenarios). Participants were presented with each patient scenario and asked to complete the MMApp. In order to benchmark the time required to use the MMApp against a commonly used clinical tool, participants were also instructed to apply each scenario patient to the American College of Surgeons National Surgical Quality Improvement Program Surgical Risk Calculator (ACS-NSQIP-SRC) [29]. Participants were instructed to select Exploratory Laparotomy with CPT 49000 as the procedure of choice for all patients when using the ACS-NSQIP-SRC and to fill in all other data points using only the information offered in the hypothetical patient’s History and Physical notes. This was done on a computer using the ACS NSQIP website [29].

#### Recruitment

To amass 50 total trials of MMApp (using 5 hypothetical scenarios), a total of 10 surgical residents from a single academic, tertiary care hospital were recruited for participation in feasibility testing using convenience sampling. None of the recruited surgical residents were part of the app development team in any way. Residents were contacted through text message to request participation and were not compensated. All included participants were in good academic standing at the institution involved and at least in postgraduation year 2 (PGY-2) or above. The study institution has a 7-year surgical residency (PGY-1 through PGY-7), with research years taken during PGY-4 and PGY-5. PGY-1 residents were excluded as they may not have adequate clinical experience with the initial evaluation of EGS patients.

#### Analysis

The time to complete MMApp and ACS-NSQIP-SRC (in seconds) for each scenario was recorded by a stopwatch, controlled by a study team member administering the questionnaire and trial of MMApp (CBR). Time to complete each scenario with both MMApp and the ACS NSQIP-SRC was averaged across all participants and compared using paired *t* tests. For MMApp, comorbidities selected for each scenario were recorded, along with the number of comorbidities correctly chosen, incorrectly chosen, and missed for each scenario. The sensitivity and specificity of identifying a patient as multimorbid using MMApp were calculated using composite data from all scenarios. Usability and satisfaction with MMApp were assessed using an 18-item questionnaire administered immediately after completing all 5 scenarios, adapted from similar surveys used to assess mobile apps (see Section 4 in Multimedia Appendix 1 for the questionnaire) [30,31]. Participants rated agreement or disagreement with each questionnaire statement on a 5-point Likert-type scale: strongly disagree (1 point), disagree (2 points), neutral (3 points), agree (4 points), and strongly agree (5 points). The mean response score and SD across all participants were calculated. Open feedback from participants was clustered into themes of positive feedback and negative or formative feedback and analyzed for trends and comparison.

### Ethical Considerations

This study was deemed exempt from review by the institutional review board at the University of Pennsylvania (protocol number 850891). Verbal consent for participation was obtained at the time of the study, allowing for both data collection and secondary analysis of research data, as approved by the institutional review board. All study data was deidentified at the time of collection before storage or analysis. Subjects were not compensated for their participation.

## Results

### Participant Demographics

There were 10 participants in this first-phase feasibility trial of the MMApp, all current resident physicians in general surgery at a single academic medical center, each of whom used the MMApp and the ACS-NSQIP-SRC for 5 scenarios (for a total of 50 trials). Of the participants, half (n=5) identified as female and half (n=5) identified as male (Table 1). Most participants were midlevel residents between PGY-4 (n=4) and PGY-5 (n=2). Beyond that, there was 1 participant from PGY-2, PGY-3, PGY-6, and PGY-7. All participants had already completed 1 month of night shifts as the primary resident physician seeing new general surgery consults, and all but 1 participant (9 total) had already completed 1 month of day shifts in this role.

**Table 1:** Participant demographics.

Demographics		Participants, n (%)
Females		5 (50)
**Postgraduation year (years since medical school graduation)**		
	2	1 (10)
	3	1 (10)
	4	4 (40)
	5	2 (20)
	6	1 (10)
	7	1 (10)
**Consult experience (primary resident physician receiving new general surgery consults)**		
	1+ months of day shifts	9 (90)
	1+ months of night shifts	10 (100)

**Time to App Completion**

The time to complete MMApp and the ACS-NSQIP-SRC varied by patient scenario (Table 2). For scenarios A and C, there was no significant difference in the time it took to complete MMApp or ACS-NSQIP-SRC (Scenario A: 86.3 seconds vs 74.3 seconds, *P*=.85. Scenario C: 58.4 seconds vs 68.8 seconds, *P*=.06). MMApp took more time to complete than ACS-NSQIP-SRC for scenario D (78.8 seconds vs 58.5 seconds, *P*=.02) and less time than ACS-NSQIP-SRC for scenarios B (76.1 seconds vs 87.4 seconds, *P*=.03) and E (20.7 seconds vs 73 seconds, *P*<.001).

**Table 2:** Mean time to complete MMApp^a^ and ACS NSQIP^b^ surgical risk calculator.

Scenario	MMApp time (seconds)	ACS NSQIP time (seconds)	*P* value
A	86.3	74.3	.85
B	76.1	87.4	.03
C	58.4	68.9	.06
D	78.8	58.5	.02
E	20.7	73.0	*<*.001

^a^MMApp: Multimorbid Patient Identifier App.

^b^ACS NSQIP: American College of Surgeons National Surgical Quality Improvement Program.

### MMApp Selections

The MMApp identified multimorbidity with 96.7% (22/30) sensitivity and 95% (19/20) specificity (see Section 6 in Multimedia Appendix 1, which shows these calculations and associated data). Of 50 total trials, during 3 trials (6%) a comorbid condition was incorrectly selected; that is, the condition was not present in the scenario but was chosen by the participant (Table 3). This occurred in scenario A among 2 participants (1 participant incorrectly selected “endocrine/metabolic disorder” and 1 participant incorrectly selected “acute kidney injury”) and in scenario B with 1 participant (incorrectly selecting “substance abuse”). There were no instances of more than one comorbidity being incorrectly selected for a given scenario. Of 50 total trials, there were 19 (38%) instances in which at least one comorbidity was missed (ie, not selected when it should have been) and 6 instances in which 2 comorbidities were missed. This occurred in scenario A (2 participants missed “cardiac arrhythmia”), scenario B (9 participants missed “protein-calorie malnutrition,” 3 participants missed “depression,” and 1 participant missed “chronic lung disease”), scenario C (3 participants missed “acute kidney injury”), and scenario E (6 participants missed “pneumonia” and 3 participants missed “substance abuse”). There were no instances when more than two comorbidities were missed.

**Table 3:** MMApp (Multimorbid Patient Identifier App) selections.

Metric		Scenario					Total
		A	B	C	D	E	
**Scenario characteristics**							
	Multimorbid (yes or no)	No	Yes	Yes	No	Yes	
	Number of intended comorbidities	2	4	3	2	2	13
	Number of total possible comorbidities	2	5	4	2	4	17
Participants who correctly identified multimorbidity status, n (%)		9 (90)	9 (90)	10 (100)	10 (100)	10 (100)	48 (96)
**Participants who incorrectly chose, n (%)**							
	1 Comorbidity	2 (20)	1 (10)	0 (0)	0 (0)	0 (0)	3 (6)
	>1 Comorbidity	0 (0)	0 (0)	0 (0)	0 (0)	0 (0)	0 (0)
**Participants who missed selection of, n (%)**							
	1 Comorbidity	2 (20)	9 (90)	3 (30)	0 (0)	6 (60)	19 (38)
	>1 Comorbidities	0 (0)	3 (30)	0 (0)	0 (0)	3 (30)	6 (12)
	>2 Comorbidities	0 (0)	0 (0)	0 (0)	0 (0)	0 (0)	0 (0)

### MMApp User Feedback

Participants strongly agreed that MMApp was easy to use (mean rating 4.8, SD 0.42) and had a sufficient introduction (mean rating 4.7, SD 0.48; Table 4). Participants also agreed or strongly agreed that MMApp required an acceptable amount of time (mean rating 4.5, SD 0.71), was intuitive (mean rating 4.5, SD 0.52), that they would use MMApp if it included risk information (mean rating 4.5, SD 0.71), and that they would recommend its use to others (mean rating 4.5, SD 0.53). Participants disagreed that MMApp was boring (mean rating 1.9, SD 0.88), required more training to use (mean 1.6, SD 0.52), was too time-consuming (mean rating 1.7, SD 0.48), or would be distracting from work or patient care (mean rating 1.5, SD 0.53).

Regarding MMapp open feedback (Table 5), participants noted that MMApp was easy to use and got easier with repeated use. They liked that it terminated once a patient was identified as multimorbid and found the design and organization to be intuitive. One participant even noted they “could use it on their phone on the walk back from the emergency department.” Participants did note that for certain conditions, especially “diabetes with complications,” they were unsure of how to appropriately select comorbidities. Participants noted that MMApp would likely be helpful if it provided prognostic information, like perioperative mortality risk, but that it would only be helpful to use clinically for patients for whom they were uncertain of the surgical risk associated with patient comorbidities.

**Table 4:** MMApp (Multimorbid Patient Identifier App) survey results.

Statement	Mean rating on a 5-point scale (1=strongly disagree; 5=strongly agree), mean (SD)	Interpretation of aggregate rating
“It was easy to use”	4.8 (0.42)	Strongly agree
“It was good to use”	4.7 (0.48)	Strongly agree
“The time spent using the app has been acceptable”	4.5 (0.71)	Agree to strongly agree
“The introduction of how to use it was sufficient”	4.7 (0.48)	Strongly agree
“I needed more training to use the app”	1.6 (0.52)	Disagree to strongly disagree
“I would recommend it to others”	4.5 (0.53)	Agree to strongly agree
“It changes the way I would think about my patient”	3.6 (0.84)	Neutral to agree
“It was too time consuming”	1.7 (0.48)	Disagree to strongly disagree
“it was boring to use”	1.9 (0.88)	Disagree
“It would be distracting from my work/patient care”	1.5 (0.53)	Disagree to strongly disagree
“It was intuitive to use”	4.5 (0.52)	Agree to strongly agree
“I would use the app if it included risk information”	4.5 (0.71)	Agree to strongly agree
“I often use a risk calculator like ACS NSQIP^a^ when evaluating patients”	1.9 (0.74)	Disagree

^a^ACS NSQIP: American College of Surgeons National Surgical Quality Improvement Program.

**Table 5:** Feedback regarding MMApp (Multimorbid Patient Identifier App).

Feedback realm		Excerpted quotes
Comparing MMApp and ACS NSQIP^a^ Surgical Risk Calculator		PA50: “(MMApp) is way faster than NSQIP” PA80: “I think (MMApp) was easy to use, much easier than NSQIP and I see why I don’t use (NSQIP)” PA70: “I worry that (NSQIP) underestimates (Patient E)’s risk” PA11: “I’m bad at remembering ASAb classes”
**Positive feedback**		
	Overall	PA20: “Really easy to use” PA60: “It gets easier (to use) with repeated use”
	Regarding features	PA10: “Once you reach (that a patient is multimorbid) it stops and you don’t need to keep clicking” PA30: “I like that it terminates once multimorbidity is established” PA70: “It was beautiful! Very user friendly. It’s awesome that it cuts out once the threshold hits”
	Regarding organization	PA30: “Navigation organized by systems with easy to click buttons” PA90: “Identifying morbidities by systems was intuitive” PA40: “Quick, clear descriptions of what qualified for each comorbidity” PA11: “Good interface, easy to advance through skills” PA80: “Seamless to use, well defined app. Could use it on my phone on the walk back from the Emergency Department”
**Negative or formative feedback**		
	Regarding selections	PA40: “Some (sections) aren’t clear like ‘diabetes with complications’. What about ‘diabetes without complications’?” PA90: “There were some listed conditions that I wasn’t sure my patient qualified for” PA11: “Criteria for some categories, like diabetes or substance abuse, were confusing”
	Regarding utility	PA30: “At the end, when it says a patient has multimorbidity, it should tell you how to interpret that information for that patient and procedure” PA80: “I think its utility will (hinge) on the clinical significance of being multimorbid” PA90: “Is there a way to give a percentage risk for perioperative mortality or morbidity?” PA80: “I think its utility will (hinge) on the clinical significance of being multimorbid (…) and how much that adds beyond intuition. I don’t need an app to tell me something is high risk for a patient with septic shock. Maybe with no medical or surgical history for an elective case, I don’t need an app to tell me they are low risk. I would use it when I have questions or uncertainty about how high risk they are. If so, the granularity would be nice to have”

^a^ACS NSQIP: American College of Surgeons National Surgical Quality Improvement Program.

^b^ASA: American Society of Anesthesiologists.

## Discussion

### Principal Results

In this first-stage feasibility study of MMApp, we found MMApp to be reasonable for use in the EGS setting. We say this as (1) resident perception of app use was overall positive, especially with regard to app usability (satisfaction) and required time for use (efficiency), and (2) MMApp did not take substantially more time to complete than the ACS-NSQIP-SRC, with mean user times well under 2 minutes. Furthermore, MMApp was able to identify multimorbidity (ie, presence of a Qualifying Comorbidity Set) from the History and Physical note of a hypothetical EGS patient with high sensitivity and specificity, suggesting effective operationalization of a claims-based definition to be able to use in prospective clinical investigations. Finally, the success of this type of app, along with positive user feedback, suggests that this type of mobile app could serve as a model for future investigation teams considering efficient methods to screen potential participants for research enrollment.

### Comparison With Previous Work

#### Defining Multimorbidity: Why an App to Identify Qualifying Comorbidity Sets

More than 50% of EGS patients are over 60 years old, and one-third are older than 70 years [32], many with multiple medical problems and at high risk for adverse outcomes [33-35]. However, the best and most efficient way to determine if an older patient is “high risk” in the clinical setting is yet to be clearly determined. Multimorbidity, defined by the presence of a Qualifying Comorbidity Set, has been used in retrospective, claims-based analyses to identify patients at particularly high risk for adverse outcomes after EGS [11,12], but translation for easy clinical use and validation had not been possible before the creation of MMApp. Ho et al [36] found functional limitations to be a very important risk factor for long-term survival after admission for an EGS condition. Frailty, though recognized to be associated with adverse clinical outcomes, has no gold-standard definition [13] and the frailty phenotype requires physical assessment of patients’ baseline clinical status, like grip strength, endurance, and walking speed [14], which can be difficult in an emergency setting or with an acutely ill patient. In that vein, Qualifying Comorbidity Sets, and thus MMApp should importantly include functional status indicators, like home oxygen or wheelchair use, which could serve as indicators of both functional limitation and frailty as contributing factors toward multimorbidity. Furthermore, a large prospective cohort study in the United Kingdom has found multimorbidity to be associated with a higher risk of mortality than frailty or disability [15]. Future work is necessary toward the validation of MMApp in further trials and could include a direct comparison of MMApp to frailty-based apps to predict the risk of adverse outcomes.

#### MMApp Usability

MMApp was designed for ease of use. In this study, most participants reported a disinterest in using the ACS-NSQIP-SRC [29] when evaluating EGS patients, in part due to the detailed information required for use, which can be difficult to find in the chart at the time of consultation (eg, the American Society of Anesthesiologists [ASA] classification). Alternative tools, such as the Emergency General Surgery Frailty Index (EGSFI), also require information that is difficult to find in the chart regarding the patient’s baseline functional status (eg, whether the patient needs assistance with specific activities of daily living, including toileting, housework, and grooming) [16]. As such, it is important to limit the amount of time, effort, and information input required by the clinician trying to determine if their patient is at a particularly high risk for adverse outcomes. MMApp uses information easily found in the patient’s medical record, standard history, and physical examination. In the EGS setting, we have demonstrated multimorbidity to be a comprehensive mechanism to stratify risk among EGS patients [11,12]. Based on this study’s findings, MMApp can be used to efficiently translate clinical findings into a diagnosis of multimorbidity, meriting future validation and investigation.

#### Health Equity

Multimorbidity is found in a higher proportion of ethnic minority groups [17] and is earlier in onset among non-Hispanic Black American than among non-Hispanic White individuals [18]. These disparate patterns of multimorbidity have been identified by the NIH as an area of interest [6]. The use of MMApp has the potential to advance health equity by allowing clinicians to easily classify patients as multimorbid. Given that non-Hispanic Black patients have lower participation rates in clinical trials [19-21] and higher rates of mortality and complications after EGS than White patients [22,23], it is important to understand the impact of multimorbidity on these disparities.

#### Generalization

The efficient patient population identification made possible by MMApp could be generalized to other research and clinical settings. This could include facilitation of eventual risk prediction and goal setting for treatment plans or recruitment for clinical trials. Streamlined recruitment is especially important when trying to enroll subjects who are not easy to “see” or when there is complexity in the exclusion or inclusion criteria. Mobile apps are now being used and studied at all levels of research, from identifying subclinical atrial fibrillation in at-risk populations [24], to streamlined questionnaire administration for patient use [25], to at-home self-assessments to improve patient participation in interventions outside of the clinical setting [26]. However, many of these mobile apps are aimed toward patient use. Beyond MMApp itself, a simple, physician-facing app to quickly screen patients for enrollment in prospective clinical trials could boost invitations for participation, especially in the emergency setting when there is limited time. The methodology used to generate MMApp could be applied to generate similar apps to be used by research teams aiming to increase the efficiency of study recruitment.

### Limitations

This study is not without limitations. To start, the scenarios for input into MMApp and the ACS-NSQIP-SRC are hypothetical in nature and certainly do not encompass all possible acute and chronic diagnoses within EGS or general surgery. Furthermore, participants were only able to use a written History and Physical to garner patient information instead of patient interviews, laboratories, or additional documentation within the electronic medical record, etc. This could potentially increase the amount of time a clinician would spend thinking through MMApp input but would likely have similar effects to other app usage (like the ACS-NSQIP-SRC). This trial was not designed to prove the superiority of MMApp to any other clinician tool, including the ACS-NSQIP-SRC. This trial was performed at only 1 institution, which could limit the generalizability of our findings. However, we believe that our findings support the feasibility of MMApp for future investigation and use in the EGS setting. Future work will validate the use of MMApp and compare it to other markers of patient risk. Including frailty will be beneficial and necessary before true clinical adaptation and prognostication through MMApp.

### Conclusions

MMApp is a convenient tool to identify multimorbidity among patients and is feasible for further validation even in the EGS setting. This will allow a prospective investigation of multimorbidity among EGS patients. At this point, MMApp is purely investigational, but the results of this study support continued investigation, validation, and eventual publication of this app. Furthermore, this type of mobile app could serve as a template for other research teams to create a tool to easily screen participants for potential enrollment.

## Appendix

Multimedia Appendix 1 This multimedia appendix includes comprehensive and complete information regarding both the claims-based definitions and the clinical definitions of comorbid conditions that make up any Qualifying Comorbidity Set. This supplement also includes screenshots to display and explain the progression through the Multimorbid Patient Identifier App (MMApp) for both multimorbid and non-multimorbid patients, hypothetical patient scenarios and research preamble used in the feasibility study, the 18-item questionnaire completed by participants regarding app usability, and the sensitivity and specificity calculations for multimorbidity identification with MMApp.

## Abbreviations

ACS-NSQIP-SRC: American College of Surgeons National Surgical Quality Improvement Program Surgical Risk Calculator
ASA: American Society of Anesthesiologists
CMS: Centers for Medicare and Medicaid
CPT: Current Procedural Terminology
EGS: emergency general surgery
EGSFI: Emergency General Surgery Frailty Index
HCC: Medicare Hierarchical Condition Category
HCPCS: Health Care Common Procedure Coding System
ICD 9/10-CM: International Classification of Disease Ninth/Tenth Revision Clinical Modification
MMApp: Multimorbid Patient Identifier App
NIH: National Institutes of Health
PGY: postgraduation year

## Data Availability

Data sharing is not applicable to this study as no data sets were generated or analyzed during this study.
