# Supplementary material for: A Novel Mobile App to Identify Patients With Multimorbidity in the Emergency Setting: Development of an App and Feasibility Trial
Source: JMIR Form Res. 2023 Jul 13;7:e42970. doi: 10.2196/42970 (PMC10375392; doi:10.2196/42970)
Supplement: Multimedia Appendix 1 [file formative_v7i1e42970_app1.docx]

Multimedia Appendix

for

**A Novel Mobile App to Identify Multimorbid Patients in the Emergency Setting: Development and Feasibility**

Claire B. Rosen, MD

Sanford E. Roberts, MD

Solomiya Syvyk, BA

Caitlin B. Finn, MD

Jason Tong, MD

Chris Wirtalla, BA

Hunter Spinks, BS

Rachel Kelz, MD MSCE MBA FACS

From the Department of Surgery, Center for Surgery and Health Economics, The University of Pennsylvania Perelman School of Medicine, Philadelphia, PA (CBR, SER, SS, CBF, JT, CW, HS, RRK).

**With instrumental support from, and appreciation for**

**The Center for Outcomes Research at the Children’s Hospital of Philadelphia Research Institute,** Philadelphia, PA, led by **Jeffrey H. Silber, MD, PhD** for **identification of comorbid conditions** with International Classification of Disease (ICD), Current Procedural Terminology (CPT) and Healthcare Common Procedure Coding System (HCPCS) Codes and for **identification/creation of Qualifying Comorbidity Sets.**

**Table of Contents**

| **Section 1: Conversion of Multimorbid Definition from Claims-Based to Clinical………………………** | p.3 |
| --- | --- |
|  |  |
| Table S1: Silber Billing Codes used to identify Comorbid Conditions, by Version 9 and 10 International Classification of Disease (ICD), Current Procedural Terminology (CPT), and Healthcare Common Procedure Coding System (HCPCS) Codes……………………………………………………............................. | p.3 |
|  |  |
| Table S2: Silber Qualifying Comorbidity Sets comprised of single, double, and triple combinations…………. | p.22 |
|  |  |
| Table S3: Clinical Description of Comorbid Conditions for Input into The Multimorbid Patient Identifier App (MMApp)…………………………………………………………………………………………………………………. | p.24 |
|  |  |
| **Section 2: MMApp Screenshots**…………………………………………………………………………………... | p.26 |
|  |  |
| Table S4: MMApp Screen Progression Example for Non-Multimorbid Patient (Scenario D)…………………... | p.26 |
|  |  |
| Table S5: MMApp Screen Progression Example for Multimorbid Patient (Scenario E)………………………… | p.34 |
|  |  |
| **Section 3: Hypothetical Scenarios**……………………………………………………………………………….. | p.38 |
|  |  |
| Information for Users/Preamble……………………………………………………………………………………….. | p.38 |
|  |  |
| Table S6: Abbreviation List for Hypothetical Patient Scenarios……………………………………………………. | p.38 |
|  |  |
| Hypothetical Patient Scenario A………………………………………………………………………………………. | p.40 |
|  |  |
| Hypothetical Patient Scenario B………………………………………………………………………………………. | p.41 |
|  |  |
| Hypothetical Patient Scenario C………………………………………………………………………………………. | p.42 |
|  |  |
| Hypothetical Patient Scenario D………………………………………………………………………………………. | p.43 |
|  |  |
| Hypothetical Patient Scenario E………………………………………………………………………………………. | p.44 |
|  |  |
| Comorbidities Present for Each Scenario……………………………………………………………………………. | p.45 |
|  |  |
| **Section 4:** **MMApp Survey…………………………………………………………………………………………..** | p.46 |
|  |  |
| **Section 5: Sensitivity and Specificity of Identifying Multimorbidity with MMApp………………………..** | p.47 |
|  |  |
| Table S7: Multimorbidity Identification with MMApp………………………………………………………………… | p.47 |
|  |  |
| **References………………………………………………………………………………………………………………** | p.48 |

**Section 1: Conversion of Multimorbid Definition from Claims-Based to Clinical**

**Table S1.** Silber [5] Billing Codes Used to Identify Comorbid Conditions, by Version 9 and 10 International Classification of Disease (ICD), Current Procedural Terminology (CPT), and Healthcare Common Procedure Coding System (HCPCS) Codes.

| **Comorbid Condition** | **ICD, CPT, and/or HCPCS Codes** |
| --- | --- |
| Acute Myocardial Infarction | ICD-9 codes: 41001, 41011, 41021, 41031, 41041, 41051, 41061, 41071, 41081, 41091, 4295-4296  ICD-10 codes: I2101-I2102, I2109, I2111, I2119, I2121, I2129, I213-I214, I219, I21A1, I21A9-I222, I228-I229, I234-I235, I511-I512 |
| Acute Renal Failure | ICD-9 codes: 5845-5849, 586  ICD-10 codes: N170-N172, N178-N179, N19 |
| Amputation and Complications | ICD-9 codes: 3536, 9059, 99760-99762, 99769, V4970-V4977, V521  ICD-10 codes: G546-G547, S48011S-S48012S, S48019S, S48021S-S48022S, S48029S, S48111S-S48112S, S48119S, S48121S-S48122S, S48129S, S48911S-S48912S, S48919S, S48921S-S48922S, S48929S, S58011S-S58012S, S58019S, S58021S-S58022S, S58029S, S58111S-S58112S, S58119S, S58121S-S58122S, S58129S, S58911S-S58912S, S58919S, S58921S-S58922S, S58929S, S68011S-S68012S, S68019S, S68021S-S68022S, S68029S, S68110S-S68129S, S68411S-S68412S, S68419S, S68421S-S68422S, S68429S, S68511S-S68512S, S68519S, S68521S-S68522S, S68529S, S68610S-S68629S, S68711S-S68712S, S68719S, S68721S-S68722S, S68729S, S78011D, S78011S-S78012D, S78012S, S78019D, S78019S, S78021D, S78021S-S78022D, S78022S, S78029D, S78029S, S78111D, S78111S-S78112D, S78112S, S78119D, S78119S, S78121D, S78121S-S78122D, S78122S, S78129D, S78129S, S78911D, S78911S-S78912D, S78912S, S78919D, S78919S, S78921D, S78921S-S78922D, S78922S, S78929D, S78929S, S88011D, S88011S-S88012D, S88012S, S88019D, S88019S, S88021D, S88021S-S88022D, S88022S, S88029D, S88029S, S88111D, S88111S-S88112D, S88112S, S88119D, S88119S, S88121D, S88121S-S88122D, S88122S, S88129D, S88129S, S88911D, S88911S-S88912D, S88912S, S88919D, S88919S, S88921D, S88921S-S88922D, S88922S, S88929D, S88929S, S98011D, S98011S-S98012D, S98012S, S98019D, S98019S, S98021D, S98021S-S98022D, S98022S, S98029D, S98029S, S98111D, S98111S-S98112D, S98112S, S98119D, S98119S, S98121D, S98121S-S98122D, S98122S, S98129D, S98129S, S98131D, S98131S-S98132D, S98132S, S98139D, S98139S, S98141D, S98141S-S98142D, S98142S, S98149D, S98149S, S98211D, S98211S-S98212D, S98212S, S98219D, S98219S, S98221D, S98221S-S98222D, S98222S, S98229D, S98229S, S98311D, S98311S-S98312D, S98312S, S98319D, S98319S, S98321D, S98321S-S98322D, S98322S, S98329D, S98329S, S98911D, S98911S-S98912D, S98912S, S98919D, S98919S, S98921D, S98921S-S98922D, S98922S, S98929D, S98929S, T8730-T8734, T8740-T8744, T8750-T8754, T8781, T8789, T879, Z44101-Z44102, Z44109, Z44111-Z44112, Z44119, Z44121-Z44122, Z44129, Z89411-Z89412, Z89419, Z89421-Z89422, Z89429, Z89431-Z89432, Z89439, Z89441-Z89442, Z89449, Z89511-Z89512, Z89519, Z89611-Z89612, Z89619 |
| Angina Pectoris | ICD-9 codes: 4130-4131, 4139  ICD-10 codes: I201, I208-I209, I25111, I25118-I25119, I25701, I25708-I25709, I25711, I25718-I25719, I25721, I25728-I25729, I25731, I25738-I25739, I25751, I25758-I25759, I25761, I25768-I25769, I25791, I25798-I25799 |
| Artificial Openings | ICD-9 codes: 53086-53087, 53640-53642, 53649, 56960-56962, 56969, 56971, 56979, V441-V444, V4450-V4452, V4459, V446, V448-V449, V551-V556, V558-V559  ICD-10 codes: K91850, K91858, K9400-K9403, K9409-K9413, K9419-K9423, K9429-K9433, K9439, Z431-Z436, Z438-Z439, Z931-Z934, Z9350-Z9352, Z9359, Z936, Z938-Z939 |
| Asthma | ICD-9 codes: 49300-49302, 49310-49312, 49381-49382, 49390-49392  ICD-10 codes: J4520-J4522, J4530-J4532, J4540-J4542, J4550-J4552, J45901-J45902, J45909, J45990-J45991, J45998 |
| Cancers, Other | ICD-9 codes: 1410-1416, 1418-1422, 1428-1431, 1438-1441, 1448-1456, 1458-1473, 1478-1483, 1488-1491, 1498-1499, 1530-1543, 1548, 1590-1591, 1598-1605, 1608-1613, 1618-1619, 1640-1643, 1648-1650, 1658-1659, 1700-1710, 1712-1729, 1740-1746, 1748-1750, 1759-1765, 1768-1769, 179, 1800-1801, 1808-1809, 181, 1820-1821, 1828, 1830, 1832-1835, 1838-1844, 1848-1849, 185, 1860, 1869, 1871-1894, 1898-1923, 1928-1929, 193, 1940-1941, 1943-1946, 1948-1955, 1958, 1963, 1969, 1982, 19881-19882, 1991-1992, 20000-20008, 20010-20018, 20020-20028, 20030-20038, 20040-20048, 20050-20058, 20060-20068, 20070-20078, 20080-20088, 20100-20108, 20110-20118, 20120-20128, 20140-20148, 20150-20158, 20160-20168, 20170-20178, 20190-20198, 20200-20208, 20210-20218, 20220-20228, 20230-20238, 20240-20248, 20250-20258, 20260-20268, 20270-20278, 20280-20288, 20290-20298, 20410-20412, 20420-20422, 20480-20482, 20490-20492, 20810-20812, 20820-20822, 20880-20882, 20890-20892, 20900-20903, 20910-20917, 20920-20927, 20929-20936, 2250-2254, 2258-2259, 2273-2274, 22802, 2370-2371, 2373, 2375-2376, 23770-23773, 23779, 2379, 2396, 2592, 7595-7596  ICD-10 codes: C01, C020-C024, C028-C031, C039-C041, C048-C052, C058-C062, C0680, C0689, C069, C07, C080-C081, C089-C091, C098-C104, C108-C113, C118-C119, C12, C130-C132, C138-C140, C142, C148, C180-C189, C19-C20, C210-C212, C218, C260-C261, C269, C300-C301, C310-C313, C318-C323, C328-C329, C37, C380-C383, C388, C390, C399, C4000-C4002, C4010-C4012, C4020-C4022, C4030-C4032, C4080-C4082, C4090-C4092, C410-C414, C419, C430, C4310, C43111-C43112, C43121-C43122, C4320-C4322, C4330-C4331, C4339, C434, C4351-C4352, C4359-C4362, C4370-C4372, C438-C439, C460-C464, C4650-C4652, C467, C469-C470, C4710-C4712, C4720-C4722, C473-C476, C478-C479, C490, C4910-C4912, C4920-C4922, C493-C496, C498-C499, C49A0-C49A5, C49A9, C4A0, C4A10, C4A111-C4A112, C4A121-C4A122, C4A20-C4A22, C4A30-C4A31, C4A39, C4A4, C4A51-C4A52, C4A59-C4A62, C4A70-C4A72, C4A8-C4A9, C50011-C50012, C50019, C50021-C50022, C50029, C50111-C50112, C50119, C50121-C50122, C50129, C50211-C50212, C50219, C50221-C50222, C50229, C50311-C50312, C50319, C50321-C50322, C50329, C50411-C50412, C50419, C50421-C50422, C50429, C50511-C50512, C50519, C50521-C50522, C50529, C50611-C50612, C50619, C50621-C50622, C50629, C50811-C50812, C50819, C50821-C50822, C50829, C50911-C50912, C50919, C50921-C50922, C50929, C510-C512, C518-C519, C52, C530-C531, C538-C543, C548-C549, C55, C561-C562, C569, C5700-C5702, C5710-C5712, C5720-C5722, C573-C574, C577-C579, C58, C600-C602, C608-C609, C61, C6200-C6202, C6210-C6212, C6290-C6292, C6300-C6302, C6310-C6312, C632, C637-C639, C641-C642, C649, C651-C652, C659, C661-C662, C669-C681, C688-C689, C6900-C6902, C6910-C6912, C6920-C6922, C6930-C6932, C6940-C6942, C6950-C6952, C6960-C6962, C6980-C6982, C6990-C6992, C700-C701, C709-C721, C7220-C7222, C7230-C7232, C7240-C7242, C7250, C7259, C729, C73, C7400-C7402, C7410-C7412, C7490-C7492, C750-C755, C758-C763, C7640-C7642, C7650-C7652, C768, C773, C779, C792, C7981-C7982, C7A00, C7A010-C7A012, C7A019-C7A026, C7A029, C7A090-C7A096, C7A098, C7A1, C7A8, C801-C802, C8100-C8149, C8170-C8179, C8190-C8269, C8280-C8319, C8330-C8339, C8350-C8359, C8370-C8419, C8440-C8449, C8460-C8479, C8490-C8499, C84A0-C84A9, C84Z0-C84Z9, C8510-C8529, C8580-C8599, C860-C866, C882-C884, C888-C889, C9030-C9032, C9110-C9112, C9130-C9132, C9140-C9142, C9150-C9152, C9160-C9162, C9190-C9192, C91A0-C91A2, C91Z0-C91Z2, C9510-C9512, C9590-C9592, C960, C9620-C9622, C9629, C964-C966, C969, C96A, C96Z, D030, D0310, D03111-D03112, D03121-D03122, D0320-D0322, D0330, D0339, D034, D0351-D0352, D0359-D0362, D0370-D0372, D038-D039, D1802, D320-D321, D329-D334, D337, D339, D352-D354, D420-D421, D429-D434, D438-D439, D443-D447, D496, E340, Q8500-Q8503, Q8509, Q851, Q858-Q859 |
| Cancers, Severe | ICD-9 codes: 1500-1505, 1508-1516, 1518-1523, 1528-1529, 1550-1552, 1560-1562, 1568-1574, 1578-1580, 1588-1589, 1620, 1622-1625, 1628-1631, 1638-1639, 20300-20302, 20310-20312, 20380-20382, 20510-20512, 20520-20522, 20530-20532, 20580-20582, 20590-20592, 20610-20612, 20620-20622, 20680-20682, 20690-20692, 20710-20712, 20720-20722, 20780-20782  ICD-10 codes: C153-C155, C158-C166, C168-C173, C178-C179, C220-C224, C227-C229, C23, C240-C241, C248-C254, C257-C259, C33, C3400-C3402, C3410-C3412, C342, C3430-C3432, C3480-C3482, C3490-C3492, C384, C450-C452, C457, C459, C480-C482, C488, C9000-C9002, C9010-C9012, C9020-C9022, C9210-C9212, C9220-C9222, C9230-C9232, C9290-C9292, C92Z0-C92Z2, C9310-C9312, C9330-C9332, C9390-C9392, C93Z0-C93Z2, C9430-C9432, C9480-C9482 |
| Cardio and Respiratory Failure | ICD-9 codes: 42741-42742, 4275, 5184, 51851-51853, 51881-51884, 78550-78551, 7980-7982, 7989, 7991, 99801  ICD-10 codes: I462, I468-I469, I4901-I4902, J80, J810, J951-J953, J95821-J95822, J9600-J9602, J9610-J9612, J9620-J9622, J9690-J9692, R092, R570, R579, T8111XA |
| Cerebrovascular Diseases | ICD-9 codes: 09487, 34200-34202, 34210-34212, 34280-34282, 34290-34292, 3442, 34430-34432, 34440-34442, 3445, 34481, 34489, 3449, 430-431, 4320-4321, 4329, 43300-43301, 43310-43311, 43320-43321, 43330-43331, 43380-43381, 43390-43391, 43400-43401, 43410-43411, 43490-43491, 4350-4353, 4358-4359, 436, 4370-4371, 4374-4376, 4378-4379, 43820-43822, 43830-43832, 43840-43842, 43850-43853, 99702  ICD-10 codes: G450-G452, G458-G468, G8100-G8104, G8110-G8114, G8190-G8194, G830, G8310-G8314, G8320-G8324, G8330-G8334, G835, G8381-G8384, G8389, G839, I6000-I6002, I6010-I6012, I602, I6030-I6032, I604, I6050-I6052, I606-I616, I618-I619, I6200-I6203, I621, I629, I6300, I63011-I63013, I63019, I6302, I63031-I63033, I63039, I6309-I6310, I63111-I63113, I63119, I6312, I63131-I63133, I63139, I6319-I6320, I63211-I63213, I63219, I6322, I63231-I63233, I63239, I6329-I6330, I63311-I63313, I63319, I63321-I63323, I63329, I63331-I63333, I63339, I63341-I63343, I63349, I6339-I6340, I63411-I63413, I63419, I63421-I63423, I63429, I63431-I63433, I63439, I63441-I63443, I63449, I6349-I6350, I63511-I63513, I63519, I63521-I63523, I63529, I63531-I63533, I63539, I63541-I63543, I63549, I6359, I636, I6381, I6389, I639, I6501-I6503, I6509, I651, I6521-I6523, I6529, I658-I659, I6601-I6603, I6609, I6611-I6613, I6619, I6621-I6623, I6629, I663, I668-I669, I672, I675-I677, I6781-I6782, I67841, I67848, I67850, I67858, I6789, I679-I680, I682, I688, I69031-I69034, I69039, I69041-I69044, I69049, I69051-I69054, I69059, I69061-I69065, I69069, I69131-I69134, I69139, I69141-I69144, I69149, I69151-I69154, I69159, I69161-I69165, I69169, I69231-I69234, I69239, I69241-I69244, I69249, I69251-I69254, I69259, I69261-I69265, I69269, I69331-I69334, I69339, I69341-I69344, I69349, I69351-I69354, I69359, I69361-I69365, I69369, I69831-I69834, I69839, I69841-I69844, I69849, I69851-I69854, I69859, I69861-I69865, I69869, I69931-I69934, I69939, I69941-I69944, I69949, I69951-I69954, I69959, I69961-I69965, I69969, I97810-I97811, I97820-I97821 |
| Chronic Kidney Disease, Stage 1-3 and Nephritis | ICD-9 codes: 0786, 40300, 40310, 40390, 40400-40401, 40410-40411, 40490-40491, 5800, 5804, 58081, 58089, 5809-5813, 58181, 58189, 5819-5822, 5824, 58281, 58289, 5829-5832, 5834, 5836-5837, 58381, 58389, 5839, 5851-5853, 5859, 75314  ICD-10 codes: A3684, A985, B520, D8684, E0821-E0822, E0829, E0921-E0922, E0929, E1021-E1022, E1029, E1121-E1122, E1129, E1321-E1322, E1329, I129-I130, I1310, M3214-M3215, M3504, N000-N079, N08, N140-N144, N150, N158, N181-N183, N189, Q6111, Q6119 |
| Chronic Kidney Disease, Stage 4-5 and Dialysis | ICD-9 codes: 40301, 40311, 40391, 40402-40403, 40412-40413, 40492-40493, 5854-5856, 99656, 99668, 99673, V4511-V4512, V560-V562, V5631-V5632, V568  ICD-10 codes: I120, I1311, I132, N184-N186, T81502A, T81502D, T81502S, T81512A, T81512D, T81512S, T81522A, T81522D, T81522S, T81532A, T81532D, T81532S, T81592A, T81592D, T81592S, T8241XA, T8241XD, T8241XS-T8242XA, T8242XD, T8242XS-T8243XA, T8243XD, T8243XS, T8249XA, T8249XD, T8249XS, T85611A, T85611D, T85611S, T85621A, T85621D, T85621S, T85631A, T85631D, T85631S, T85691A, T85691D, T85691S, T8571XA, T8571XD, T8571XS, Y622, Z4901-Z4902, Z4931-Z4932, Z9115, Z992 |
| Chronic Lung Diseases | ICD-9 codes: 135, 27700-27703, 27709, 4910-4911, 49120-49122, 4918-4920, 4928, 49320-49322, 4940-4941, 4950-4959, 496, 500-505, 5060-5064, 5069, 5080-5082, 5088-5089, 515, 5160-5162, 51630-51637, 5164-5165, 51661-51664, 51669, 5168-5169, 5171-5172, 5178, 5181-5183, 5186  ICD-10 codes: B4481, D860, D862, E840, E8411, E8419, E848-E849, J410-J411, J418, J42, J430-J432, J438-J441, J449, J470-J471, J479, J60-J61, J620, J628, J630-J636, J64-J65, J660-J662, J668, J670-J684, J688-J689, J700-J705, J708-J709, J82, J8401-J8403, J8409-J8410, J84111-J84117, J8417, J842, J8481-J8483, J84841-J84843, J84848, J8489, J849, J982-J983, J99, M3213, M3301, M3311, M3321, M3391, M3481, M3502 |
| Chronic Pancreatitis | ICD-9 codes: 5771  ICD-10 codes: K860-K861 |
| Chronic Ulcer, Skin, Not Pressure | ICD-9 codes: 70710-70715, 70719, 7078-7079  ICD-10 codes: E08621-E08622, E09621-E09622, E10621-E10622, E11621-E11622, E13621-E13622, I70231-I70235, I70238-I70239, I70241-I70245, I70248-I70249, I7025, I70331-I70335, I70338-I70339, I70341-I70345, I70348-I70349, I7035, I70431-I70435, I70438-I70439, I70441-I70445, I70448-I70449, I7045, I70531-I70535, I70538-I70539, I70541-I70545, I70548-I70549, I7055, I70631-I70635, I70638-I70639, I70641-I70645, I70648-I70649, I7065, I70731-I70735, I70738-I70739, I70741-I70745, I70748-I70749, I7075, L97101-L97106, L97108-L97109, L97111-L97116, L97118-L97119, L97121-L97126, L97128-L97129, L97201-L97206, L97208-L97209, L97211-L97216, L97218-L97219, L97221-L97226, L97228-L97229, L97301-L97306, L97308-L97309, L97311-L97316, L97318-L97319, L97321-L97326, L97328-L97329, L97401-L97406, L97408-L97409, L97411-L97416, L97418-L97419, L97421-L97426, L97428-L97429, L97501-L97506, L97508-L97509, L97511-L97516, L97518-L97519, L97521-L97526, L97528-L97529, L97801-L97806, L97808-L97809, L97811-L97816, L97818-L97819, L97821-L97826, L97828-L97829, L97901-L97906, L97908-L97909, L97911-L97916, L97918-L97919, L97921-L97926, L97928-L97929, L98411-L98416, L98418-L98419, L98421-L98426, L98428-L98429, L98491-L98496, L98498-L98499 |
| Congestive Heart Failure | ICD-9 codes: 39891, 40201, 40211, 40291, 4150, 4160-4161, 4168-4171, 4178-4179, 4250, 42511, 42518, 4252-4255, 4257-4259, 4280-4281, 42820-42823, 42830-42833, 42840-42843, 4289-4291  ICD-10 codes: A3681, B3324, I0981, I110, I130, I132, I2601-I2602, I2609, I270-I271, I2720-I2724, I2729, I2781, I2783, I2789, I279-I281, I288-I289, I420-I429, I43, I501, I5020-I5023, I5030-I5033, I5040-I5043, I50810-I50814, I5082-I5084, I5089, I509, I514-I515 |
| Coronary Artery Disease | ICD-9 codes: 412, 41400-41407, 41410-41412, 41419, 4142-4144, 4148-4149, 74685  ICD-10 codes: I2510, I252-I253, I2541-I2542, I255-I256, I25810-I25812, I2582-I2584, I2589, I259, Q245 |
| Depression, Other | ICD-9 codes: 3004, 30110-30113, 3091, 311  ICD-10 codes: F3281, F3289, F329, F340-F341, F530 |
| Diabetes with Complications | ICD-9 codes: 24910-24911, 24920-24921, 24930-24931, 24940-24941, 24950-24951, 24960-24961, 24970-24971, 24980-24981, 24990-24991, 25010-25013, 25020-25023, 25030-25033, 25040-25043, 25050-25053, 25060-25063, 25070-25073, 25080-25083, 25090-25093, 3572, 36201, 36203-36207, 36641  ICD-10 codes: E0800-E0801, E0810-E0811, E0821-E0822, E0829, E08311, E08319, E083211-E083213, E083219, E083291-E083293, E083299, E083311-E083313, E083319, E083391-E083393, E083399, E083411-E083413, E083419, E083491-E083493, E083499, E083511-E083513, E083519, E083521-E083523, E083529, E083531-E083533, E083539, E083541-E083543, E083549, E083551-E083553, E083559, E083591-E083593, E083599, E0836, E0837X1-E0837X3, E0837X9, E0839-E0844, E0849, E0851-E0852, E0859, E08610, E08618, E08620-E08622, E08628, E08630, E08638, E08641, E08649, E0865, E0869, E088, E0900-E0901, E0910-E0911, E0921-E0922, E0929, E09311, E09319, E093211-E093213, E093219, E093291-E093293, E093299, E093311-E093313, E093319, E093391-E093393, E093399, E093411-E093413, E093419, E093491-E093493, E093499, E093511-E093513, E093519, E093521-E093523, E093529, E093531-E093533, E093539, E093541-E093543, E093549, E093551-E093553, E093559, E093591-E093593, E093599, E0936, E0937X1-E0937X3, E0937X9, E0939-E0944, E0949, E0951-E0952, E0959, E09610, E09618, E09620-E09622, E09628, E09630, E09638, E09641, E09649, E0965, E0969, E098, E1010-E1011, E1021-E1022, E1029, E10311, E10319, E103211-E103213, E103219, E103291-E103293, E103299, E103311-E103313, E103319, E103391-E103393, E103399, E103411-E103413, E103419, E103491-E103493, E103499, E103511-E103513, E103519, E103521-E103523, E103529, E103531-E103533, E103539, E103541-E103543, E103549, E103551-E103553, E103559, E103591-E103593, E103599, E1036, E1037X1-E1037X3, E1037X9, E1039-E1044, E1049, E1051-E1052, E1059, E10610, E10618, E10620-E10622, E10628, E10630, E10638, E10641, E10649, E1065, E1069, E108, E1100-E1101, E1110-E1111, E1121-E1122, E1129, E11311, E11319, E113211-E113213, E113219, E113291-E113293, E113299, E113311-E113313, E113319, E113391-E113393, E113399, E113411-E113413, E113419, E113491-E113493, E113499, E113511-E113513, E113519, E113521-E113523, E113529, E113531-E113533, E113539, E113541-E113543, E113549, E113551-E113553, E113559, E113591-E113593, E113599, E1136, E1137X1-E1137X3, E1137X9, E1139-E1144, E1149, E1151-E1152, E1159, E11610, E11618, E11620-E11622, E11628, E11630, E11638, E11641, E11649, E1165, E1169, E118, E1300-E1301, E1310-E1311, E1321-E1322, E1329, E13311, E13319, E133211-E133213, E133219, E133291-E133293, E133299, E133311-E133313, E133319, E133391-E133393, E133399, E133411-E133413, E133419, E133491-E133493, E133499, E133511-E133513, E133519, E133521-E133523, E133529, E133531-E133533, E133539, E133541-E133543, E133549, E133551-E133553, E133559, E133591-E133593, E133599, E1336, E1337X1-E1337X3, E1337X9, E1339-E1344, E1349, E1351-E1352, E1359, E13610, E13618, E13620-E13622, E13628, E13630, E13638, E13641, E13649, E1365, E1369, E138 |
| Diabetes, No Complications | ICD-9 codes: 24900-24901, 25000-25003, V5867  ICD-10 codes: E089, E099, E109, E119, E139, Z794 |
| Disorders of Immunity | ICD-9 codes: 27900-27906, 27909-27913, 27919, 2792-2793, 27941, 27949-27953, 2798-2799, 28411-28412, 28419, 28800-28804, 28809, 2881-2882, 2884  ICD-10 codes: D61810-D61811, D61818, D700-D704, D708-D709, D71, D720, D761-D763, D800-D813, D8130-D8132, D8139, D814-D817, D8189, D819-D824, D828-D832, D838-D840, D848-D849, D893, D8940-D8943, D8949, D89810-D89813, D8982, D8989, D899 |
| Endocrine and Metabolic Disorders | ICD-9 codes: 0363, 2510, 25200-25202, 25208, 2521, 2528-2541, 2548-2550, 25510-25514, 2552-2553, 25541-25542, 2555-2556, 2558-2559, 25801-25803, 2581, 2588-2589, 2700-2711, 2714, 2718-2719, 2727, 2732-2734, 27501, 2771-2772, 27730-27731, 27739, 2775-2776, 27781-27787, 27789, 5881, 58881  ICD-10 codes: A391, C880, D841, D891, E035, E15, E200, E208-E215, E220-E222, E228-E233, E236-E237, E240-E244, E248-E250, E258-E259, E2601-E2602, E2609, E261, E2681, E2689, E269-E273, E2740, E2749, E275, E278-E279, E310-E311, E3120-E3123, E318-E321, E328-E329, E344, E700-E701, E7020-E7021, E7029-E7030, E70310-E70311, E70318-E70321, E70328-E70331, E70338-E70339, E7039-E7041, E7049, E705, E708-E710, E71110-E71111, E71118, E71120-E71121, E71128, E7119, E712, E71310-E71314, E71318, E7132, E7139-E7143, E71440, E71448, E7150, E71510-E71511, E71518, E71520-E71522, E71528-E71529, E7153, E71540-E71542, E71548, E7200-E7204, E7209-E7212, E7219-E7223, E7229, E723-E724, E7250-E7253, E7259, E7281, E7289, E729, E7400-E7404, E7409, E7420-E7421, E7429, E744, E748-E749, E7521-E7522, E75240-E75243, E75248-E75249, E753, E7601-E7603, E761, E76210-E76211, E76219, E7622, E7629, E763, E768-E771, E778-E779, E791-E792, E798-E801, E8020-E8021, E8029, E803, E83110, E850-E854, E8581-E8582, E8589, E859, E8801, E8840-E8842, E8849, E8889, E892-E893, E896, H49811-H49813, H49819, N251, N2581 |
|  |  |
| Heart Arrhythmias | ICD-9 codes: 4260, 4270-4272, 42731-42732, 42781  ICD-10 codes: I442, I470-I472, I479-I481, I4811, I4819, I482, I4820-I4821, I483-I484, I4891-I4892, I492, I495 |
| Hematological, Other | ICD-9 codes: 2384, 23871, 23877, 23879, 2820-2823, 28243-28245, 28247, 28249, 2825, 2827-2829, 2850, 2862-2864, 28652-28653, 28659, 2866-2867, 2869-2872, 28730-28733, 28739, 2875, 2878-2879, 28981-28982, 28984  ICD-10 codes: C946, D45, D471, D473, D479, D47Z1-D47Z2, D47Z9, D550-D553, D558-D562, D564-D565, D568, D573, D580-D582, D588-D589, D640-D643, D65, D680-D682, D68311-D68312, D68318, D6832, D684, D6851-D6852, D6859, D6861-D6862, D6869, D688-D693, D6941-D6942, D6949, D696, D698-D699, D7582 |
| Hematological, Severe | ICD-9 codes: 23872-23876, 28241-28242, 28260-28264, 28268-28269, 2830, 28310-28311, 28319, 2832, 2839, 28401, 28409, 2842, 28481, 28489, 2849, 2860-2861, 28952, 28983, 5173  ICD-10 codes: D460-D461, D4620-D4622, D464, D469, D46A, D46B, D46C, D46Z, D474, D5700-D5702, D571, D5720, D57211-D57212, D57219, D5740, D57411-D57412, D57419, D5780, D57811-D57812, D57819, D590-D596, D598-D601, D608-D609, D6101, D6109, D611-D613, D6182, D6189, D619, D66-D67, D7581 |
| HIV/AIDS | ICD-9 codes: 042, 07953, V08  ICD-10 codes: B20, B9735, Z21 |
| Hypertension | ICD-9 codes: 4010-4011, 4019, 40200, 40210, 40290, 40501, 40509, 40511, 40519, 40591, 40599, 4372  ICD-10 codes: I10, I119, I150-I152, I158-I161, I169, I674, N262 |
| Implants/Grafts with Complications | ICD-9 codes: 3491, 59681-59683, 62931, 9961-9962, 99630-99631, 99639-99647, 99649, 99660-99667, 99669, 99674-99678  ICD-10 codes: M96621-M96622, M96629, M96631-M96632, M96639, M9665, M96661-M96662, M96669, M96671-M96672, M96679, M9669, N99510-N99512, N99518, N99520-N99524, N99528, N99530-N99534, N99538, T82310A-T82312A, T82318A-T82322A, T82328A-T82332A, T82338A-T82339A, T82390A-T82392A, T82398A-T82399A, T82510A-T82511A, T82513A-T82515A, T82518A, T82520A-T82521A, T82523A-T82525A, T82528A, T82530A-T82531A, T82533A-T82535A, T82538A, T82590A-T82591A, T82593A-T82595A, T82598A, T826XXA-T827XXA, T82818A, T82828A, T82838A, T82848A, T82856A, T82858A, T82868A, T82898A, T83010A-T83012A, T83018A, T83020A-T83022A, T83028A, T83030A-T83032A, T83038A, T83090A-T83092A, T83098A, T83110A-T83113A, T83118A, T83120A-T83123A, T83128A, T83190A-T83193A, T83198A, T8321XA-T8325XA, T8329XA, T83410A-T83411A, T83418A, T83420A-T83421A, T83428A, T83490A-T83491A, T83498A, T83510A-T83512A, T83518A, T83590A-T83593A, T83598A, T8361XA-T8362XA, T8369XA, T83711A-T83714A, T83718A-T83719A, T83721A-T83724A, T83728A-T83729A, T8379XA, T8381XA-T8386XA, T8389XA, T839XXA, T84010A-T84013A, T84018A-T84023A, T84028A-T84033A, T84038A-T84039A, T84050A-T84053A, T84058A-T84063A, T84068A-T84069A, T84090A-T84093A, T84098A-T84099A, T84110A-T84117A, T84119A-T84127A, T84129A, T84190A-T84197A, T84199A, T84210A, T84213A, T84216A, T84218A, T84220A, T84223A, T84226A, T84228A, T84290A, T84293A, T84296A, T84298A, T84310A, T84318A, T84320A, T84328A, T84390A, T84398A, T84410A, T84418A, T84420A, T84428A, T84490A, T84498A, T8450XA-T8454XA, T8459XA-T8460XA, T84610A-T84615A, T84619A-T84625A, T84629A, T8463XA, T8469XA, T847XXA, T8481XA-T8486XA, T8489XA, T849XXA, T8501XA-T8503XA, T8509XA, T85110A-T85113A, T85118A, T85120A-T85123A, T85128A, T85190A-T85193A, T85199A, T85615A, T85625A, T85635A, T85695A, T8572XA, T85730A-T85735A, T85738A, T8579XA, T85810A, T85820A, T85830A, T85840A, T85850A, T85860A, T85890A, T86842 |
| Inflammatory Bowel Disease | ICD-9 codes: 5550-5552, 5559-5566, 5568-5569  ICD-10 codes: K5000, K50011-K50014, K50018-K50019, K5010, K50111-K50114, K50118-K50119, K5080, K50811-K50814, K50818-K50819, K5090, K50911-K50914, K50918-K50919, K5100, K51011-K51014, K51018-K51019, K5120, K51211-K51214, K51218-K51219, K5130, K51311-K51314, K51318-K51319, K5140, K51411-K51414, K51418-K51419, K5150, K51511-K51514, K51518-K51519, K5180, K51811-K51814, K51818-K51819, K5190, K51911-K51914, K51918-K51919 |
| Lipoid Metabolism | ICD-9 codes: 2720-2725, 2728-2729  ICD-10 codes: E7130, E755-E756, E7800-E7801, E781-E783, E7841, E7849, E785-E786, E7870, E7879, E7881, E7889, E789, E882 |
| Liver Diseases | ICD-9 codes: 07022-07023, 07032-07033, 07044, 07054, 4560-4561, 45620-45621, 5712-5713, 57140-57142, 57149, 5715-5716, 5722-5724, 5728, 5735  ICD-10 codes: B180-B182, B188-B189, I8500-I8501, I8510-I8511, K7030-K7031, K7040-K7041, K7041, K709, K7111, K7201, K7210-K7211, K7290-K7291, K730-K732, K738-K739, K743-K745, K7460, K7469, K754, K766-K767, K7681 |
| Major Depressive Bipolar and Paranoid disorder | ICD-9 codes: 29600-29606, 29610-29616, 29620-29626, 29630-29636, 29640-29646, 29650-29656, 29660-29666, 2967, 29680-29682, 29689-29690, 29699, 2970-2973, 2978-2979, E9500-E9511, E9518, E9520-E9521, E9528-E9531, E9538-E9539, E954, E9550-E9557, E9559, E956, E9570-E9572, E9579-E9589, E959  ICD-10 codes: F22, F24, F3010-F3013, F302-F304, F308-F310, F3110-F3113, F312, F3130-F3132, F314-F315, F3160-F3164, F3170-F3178, F3181, F3189, F319-F325, F330-F333, F3340-F3342, F338-F339, F3481, F3489, F349, F39, T1491XA, T1491XD, T1491XS, T360X2A, T360X2S, T361X2A, T361X2S, T362X2A, T362X2S, T363X2A, T363X2S, T364X2A, T364X2S, T365X2A, T365X2S, T366X2A, T366X2S, T367X2A, T367X2S, T368X2A, T368X2S, T3692XA, T3692XS, T370X2A, T370X2S, T371X2A, T371X2S, T372X2A, T372X2S, T373X2A, T373X2S, T374X2A, T374X2S, T375X2A, T375X2S, T378X2A, T378X2S, T3792XA, T3792XS, T380X2A, T380X2S, T381X2A, T381X2S, T382X2A, T382X2S, T383X2A, T383X2S, T384X2A, T384X2S, T385X2A, T385X2S, T386X2A, T386X2S, T387X2A, T387X2S, T38802A, T38802S, T38812A, T38812S, T38892A, T38892S, T38902A, T38902S, T38992A, T38992S, T39012A, T39012S, T39092A, T39092S, T391X2A, T391X2S, T392X2A, T392X2S, T39312A, T39312S, T39392A, T39392S, T394X2A, T394X2S, T398X2A, T398X2S, T3992XA, T3992XS, T400X2A, T400X2S, T401X2A, T401X2S, T402X2A, T402X2S, T403X2A, T403X2S, T404X2A, T404X2S, T405X2A, T405X2S, T40602A, T40602S, T40692A, T40692S, T407X2A, T407X2S, T408X2A, T408X2S, T40902A, T40902S, T40992A, T40992S, T410X2A, T410X2S, T411X2A, T411X2S, T41202A, T41202S, T41292A, T41292S, T413X2A, T413X2S, T4142XA, T4142XS, T415X2A, T415X2S, T420X2A, T420X2S, T421X2A, T421X2S, T422X2A, T422X2S, T423X2A, T423X2S, T424X2A, T424X2S, T425X2A, T425X2S, T426X2A, T426X2S, T4272XA, T4272XS, T428X2A, T428X2S, T43012A, T43012S, T43022A, T43022S, T431X2A, T431X2S, T43202A, T43202S, T43212A, T43212S, T43222A, T43222S, T43292A, T43292S, T433X2A, T433X2S, T434X2A, T434X2S, T43502A, T43502S, T43592A, T43592S, T43602A, T43602S, T43612A, T43612S, T43622A, T43622S, T43632A, T43632S, T43642A, T43642S, T43692A, T43692S, T438X2A, T438X2S, T4392XA, T4392XS, T440X2A, T440X2S, T441X2A, T441X2S, T442X2A, T442X2S, T443X2A, T443X2S, T444X2A, T444X2S, T445X2A, T445X2S, T446X2A, T446X2S, T447X2A, T447X2S, T448X2A, T448X2S, T44902A, T44902S, T44992A, T44992S, T450X2A, T450X2S, T451X2A, T451X2S, T452X2A, T452X2S, T453X2A, T453X2S, T454X2A, T454X2S, T45512A, T45512S, T45522A, T45522S, T45602A, T45602S, T45612A, T45612S, T45622A, T45622S, T45692A, T45692S, T457X2A, T457X2S, T458X2A, T458X2S, T4592XA, T4592XS, T460X2A, T460X2S, T461X2A, T461X2S, T462X2A, T462X2S, T463X2A, T463X2S, T464X2A, T464X2S, T465X2A, T465X2S, T466X2A, T466X2S, T467X2A, T467X2S, T468X2A, T468X2S, T46902A, T46902S, T46992A, T46992S, T470X2A, T470X2S, T471X2A, T471X2S, T472X2A, T472X2S, T473X2A, T473X2S, T474X2A, T474X2S, T475X2A, T475X2S, T476X2A, T476X2S, T477X2A, T477X2S, T478X2A, T478X2S, T4792XA, T4792XS, T480X2A, T480X2S, T481X2A, T481X2S, T48202A, T48202S, T48292A, T48292S, T483X2A, T483X2S, T484X2A, T484X2S, T485X2A, T485X2S, T486X2A, T486X2S, T48902A, T48902S, T48992A, T48992S, T490X2A, T490X2S, T491X2A, T491X2S, T492X2A, T492X2S, T493X2A, T493X2S, T494X2A, T494X2S, T495X2A, T495X2S, T496X2A, T496X2S, T497X2A, T497X2S, T498X2A, T498X2S, T4992XA, T4992XS, T500X2A, T500X2S, T501X2A, T501X2S, T502X2A, T502X2S, T503X2A, T503X2S, T504X2A, T504X2S, T505X2A, T505X2S, T506X2A, T506X2S, T507X2A, T507X2S, T508X2A, T508X2S, T50902A, T50902S, T50912A, T50912S, T50992A, T50992S, T50A12A, T50A12S, T50A22A, T50A22S, T50A92A, T50A92S, T50B12A, T50B12S, T50B92A, T50B92S, T50Z12A, T50Z12S, T50Z92A, T50Z92S, T510X2A, T510X2S, T511X2A, T511X2S, T512X2A, T512X2S, T513X2A, T513X2S, T518X2A, T518X2S, T5192XA, T5192XS, T520X2A, T520X2S, T521X2A, T521X2S, T522X2A, T522X2S, T523X2A, T523X2S, T524X2A, T524X2S, T528X2A, T528X2S, T5292XA, T5292XS, T530X2A, T530X2S, T531X2A, T531X2S, T532X2A, T532X2S, T533X2A, T533X2S, T534X2A, T534X2S, T535X2A, T535X2S, T536X2A, T536X2S, T537X2A, T537X2S, T5392XA, T5392XS, T540X2A, T540X2S, T541X2A, T541X2S, T542X2A, T542X2S, T543X2A, T543X2S, T5492XA, T5492XS, T550X2A, T550X2S, T551X2A, T551X2S, T560X2A, T560X2S, T561X2A, T561X2S, T562X2A, T562X2S, T563X2A, T563X2S, T564X2A, T564X2S, T565X2A, T565X2S, T566X2A, T566X2S, T567X2A, T567X2S, T56812A, T56812S, T56892A, T56892S, T5692XA, T5692XS, T570X2A, T570X2S, T571X2A, T571X2S, T572X2A, T572X2S, T573X2A, T573X2S, T578X2A, T578X2S, T5792XA, T5792XS, T5802XA, T5802XS, T5812XA, T5812XS, T582X2A, T582X2S, T588X2A, T588X2S, T5892XA, T5892XS, T590X2A, T590X2S, T591X2A, T591X2S, T592X2A, T592X2S, T593X2A, T593X2S, T594X2A, T594X2S, T595X2A, T595X2S, T596X2A, T596X2S, T597X2A, T597X2S, T59812A, T59812S, T59892A, T59892S, T5992XA, T5992XS, T600X2A, T600X2S, T601X2A, T601X2S, T602X2A, T602X2S, T603X2A, T603X2S, T604X2A, T604X2S, T608X2A, T608X2S, T6092XA, T6092XS, T6102XA, T6102XS, T6112XA, T6112XS, T61772A, T61772S, T61782A, T61782S, T618X2A, T618X2S, T6192XA, T6192XS, T620X2A, T620X2S, T621X2A, T621X2S, T622X2A, T622X2S, T628X2A, T628X2S, T6292XA, T6292XS, T63002A, T63002S, T63012A, T63012S, T63022A, T63022S, T63032A, T63032S, T63042A, T63042S, T63062A, T63062S, T63072A, T63072S, T63082A, T63082S, T63092A, T63092S, T63112A, T63112S, T63122A, T63122S, T63192A, T63192S, T632X2A, T632X2S, T63302A, T63302S, T63312A, T63312S, T63322A, T63322S, T63332A, T63332S, T63392A, T63392S, T63412A, T63412S, T63422A, T63422S, T63432A, T63432S, T63442A, T63442S, T63452A, T63452S, T63462A, T63462S, T63482A, T63482S, T63512A, T63512S, T63592A, T63592S, T63612A, T63612S, T63622A, T63622S, T63632A, T63632S, T63692A, T63692S, T63712A, T63712S, T63792A, T63792S, T63812A, T63812S, T63822A, T63822S, T63832A, T63832S, T63892A, T63892S, T6392XA, T6392XS, T6402XA, T6402XS, T6482XA, T6482XS, T650X2A, T650X2S, T651X2A, T651X2S, T65212A, T65212S, T65222A, T65222S, T65292A, T65292S, T653X2A, T653X2S, T654X2A, T654X2S, T655X2A, T655X2S, T656X2A, T656X2S, T65812A, T65812S, T65822A, T65822S, T65832A, T65832S, T65892A, T65892S, T6592XA, T6592XS, T71112A, T71112S, T71122A, T71122S, T71132A, T71132S, T71152A, T71152S, T71162A, T71162S, T71192A, T71192S, T71222A, T71222S, T71232A, T71232S, X710XXA, X710XXD, X710XXS-X711XXA, X711XXD, X711XXS-X712XXA, X712XXD, X712XXS-X713XXA, X713XXD, X713XXS, X718XXA, X718XXD, X718XXS-X719XXA, X719XXD, X719XXS, X72XXXA, X72XXXD, X72XXXS, X730XXA, X730XXD, X730XXS-X731XXA, X731XXD, X731XXS-X732XXA, X732XXD, X732XXS, X738XXA, X738XXD, X738XXS-X739XXA, X739XXD, X739XXS, X7401XA, X7401XD, X7401XS-X7402XA, X7402XD, X7402XS, X7409XA, X7409XD, X7409XS, X748XXA, X748XXD, X748XXS-X749XXA, X749XXD, X749XXS, X75XXXA, X75XXXD, X75XXXS-X76XXXA, X76XXXD, X76XXXS, X770XXA, X770XXD, X770XXS-X771XXA, X771XXD, X771XXS-X772XXA, X772XXD, X772XXS-X773XXA, X773XXD, X773XXS, X778XXA, X778XXD, X778XXS-X779XXA, X779XXD, X779XXS-X780XXA, X780XXD, X780XXS-X781XXA, X781XXD, X781XXS-X782XXA, X782XXD, X782XXS, X788XXA, X788XXD, X788XXS-X789XXA, X789XXD, X789XXS, X79XXXA, X79XXXD, X79XXXS-X80XXXA, X80XXXD, X80XXXS, X810XXA, X810XXD, X810XXS-X811XXA, X811XXD, X811XXS, X818XXA, X818XXD, X818XXS, X820XXA, X820XXD, X820XXS-X821XXA, X821XXD, X821XXS-X822XXA, X822XXD, X822XXS, X828XXA, X828XXD, X828XXS, X830XXA, X830XXD, X830XXS-X831XXA, X831XXD, X831XXS-X832XXA, X832XXD, X832XXS, X838XXA, X838XXD, X838XXS |
| Major Organ Transplant | ICD-9 codes: 99681-99688, V420-V421, V426-V427, V4281-V4284, V4321-V4322  ICD-10 codes: T8600-T8603, T8609-T8613, T8619-T8623, T86290, T86298, T8630-T8633, T8639-T8643, T8649, T865, T86810-T86812, T86818-T86819, T86850-T86852, T86858-T86859, Z4821-Z4824, Z48280, Z48290, Z940-Z944, Z9481-Z9484, Z95811-Z95812 |
| Morbid Obesity | ICD-9 codes: 27801, 27803, V8541-V8545  ICD-10 codes: E6601, E662, Z6841-Z6845 |
| Neurological Disorder, Other | ICD-9 codes: 33371, 33520-33524, 33529, 340, 3410-3411, 3418-3419, 3430-3434, 3438-3439, 3570-3571, 3573-3577, 35781-35782, 35789, 3579, 35800-35801, 3581-3582, 35830-35831, 35839, 3588-3591, 35921  ICD-10 codes: D8682, G1220-G1225, G1229, G130-G131, G35, G360-G361, G368-G372, G375, G378-G379, G610-G611, G6181-G6182, G6189, G619-G622, G6281-G6282, G63, G650-G652, G7000-G7001, G701-G702, G7080-G7081, G7089, G709, G7100-G7102, G7109, G7111, G712, G731, G733, G800-G804, G808-G809, M0550, M05511-M05512, M05519, M05521-M05522, M05529, M05531-M05532, M05539, M05541-M05542, M05549, M05551-M05552, M05559, M05561-M05562, M05569, M05571-M05572, M05579, M0559, M3483 |
| Opportunistic Infections | ICD-9 codes: 0074, 0310, 0312, 0785, 1124-1125, 11284, 1173, 1175, 1177, 118, 1300, 1304, 1308, 1363, 3210, 4841, 4846  ICD-10 codes: A072, A310, A312, B250-B252, B258-B259, B371, B377, B3781, B440-B442, B447, B4489, B449-B453, B457-B465, B468-B469, B484, B488, B582-B583, B59 |
| Oxygen | CPT/HCPCS codes: E0425, E0430-E0431, E0433-E0435, E0439-E0443, E1390-E1392, K0738 |
| Paralysis | ICD-9 codes: 0522, 05314, 05474, 32302, 32342, 32352, 32363, 32372, 32382, 3340-3344, 3348-3350, 33510-33511, 33519, 3358-3363, 3368-3369, 34120-34122, 34400-34404, 34409, 3441, 34460-34461, 7400-7402, 74100-74103, 74190-74193, 7420-7424, 74251, 74253, 74259, 7428-7429, 78072, 80600-80639, 8064-8065, 80660-80662, 80669-80672, 80679, 8068-8069, 9072, 95200-95219, 9522-9524, 9528-9529  ICD-10 codes: B0082, B0112, B0224, G041, G0489, G0491, G054, G110-G114, G118-G121, G128-G129, G320, G3281, G373-G374, G8220-G8222, G8250-G8254, G834, G901, G950, G9511, G9519-G9520, G9529, G9581, G9589, G959, G992, Q000-Q002, Q010-Q012, Q018-Q019, Q02, Q030-Q031, Q038-Q046, Q048-Q064, Q068-Q069, Q0700-Q0703, Q078-Q079, R532, S140XXA, S140XXD, S140XXS, S14101A, S14101D, S14101S-S14102A, S14102D, S14102S-S14103A, S14103D, S14103S-S14104A, S14104D, S14104S-S14105A, S14105D, S14105S-S14106A, S14106D, S14106S-S14107A, S14107D, S14107S-S14108A, S14108D, S14108S-S14109A, S14109D, S14109S, S14111A, S14111D, S14111S-S14112A, S14112D, S14112S-S14113A, S14113D, S14113S-S14114A, S14114D, S14114S-S14115A, S14115D, S14115S-S14116A, S14116D, S14116S-S14117A, S14117D, S14117S-S14118A, S14118D, S14118S-S14119A, S14119D, S14119S, S14121A, S14121D, S14121S-S14122A, S14122D, S14122S-S14123A, S14123D, S14123S-S14124A, S14124D, S14124S-S14125A, S14125D, S14125S-S14126A, S14126D, S14126S-S14127A, S14127D, S14127S-S14128A, S14128D, S14128S-S14129A, S14129D, S14129S, S14131A, S14131D, S14131S-S14132A, S14132D, S14132S-S14133A, S14133D, S14133S-S14134A, S14134D, S14134S-S14135A, S14135D, S14135S-S14136A, S14136D, S14136S-S14137A, S14137D, S14137S-S14138A, S14138D, S14138S-S14139A, S14139D, S14139S, S14141A, S14141D, S14141S-S14142A, S14142D, S14142S-S14143A, S14143D, S14143S-S14144A, S14144D, S14144S-S14145A, S14145D, S14145S-S14146A, S14146D, S14146S-S14147A, S14147D, S14147S-S14148A, S14148D, S14148S-S14149A, S14149D, S14149S, S14151A, S14151D, S14151S-S14152A, S14152D, S14152S-S14153A, S14153D, S14153S-S14154A, S14154D, S14154S-S14155A, S14155D, S14155S-S14156A, S14156D, S14156S-S14157A, S14157D, S14157S-S14158A, S14158D, S14158S-S14159A, S14159D, S14159S, S240XXA, S240XXD, S240XXS, S24101A, S24101D, S24101S-S24102A, S24102D, S24102S-S24103A, S24103D, S24103S-S24104A, S24104D, S24104S, S24109A, S24109D, S24109S, S24111A, S24111D, S24111S-S24112A, S24112D, S24112S-S24113A, S24113D, S24113S-S24114A, S24114D, S24114S, S24119A, S24119D, S24119S, S24131A, S24131D, S24131S-S24132A, S24132D, S24132S-S24133A, S24133D, S24133S-S24134A, S24134D, S24134S, S24139A, S24139D, S24139S, S24141A, S24141D, S24141S-S24142A, S24142D, S24142S-S24143A, S24143D, S24143S-S24144A, S24144D, S24144S, S24149A, S24149D, S24149S, S24151A, S24151D, S24151S-S24152A, S24152D, S24152S-S24153A, S24153D, S24153S-S24154A, S24154D, S24154S, S24159A, S24159D, S24159S, S3401XA, S3401XD, S3401XS-S3402XA, S3402XD, S3402XS, S34101A, S34101D, S34101S-S34102A, S34102D, S34102S-S34103A, S34103D, S34103S-S34104A, S34104D, S34104S-S34105A, S34105D, S34105S, S34109A, S34109D, S34109S, S34111A, S34111D, S34111S-S34112A, S34112D, S34112S-S34113A, S34113D, S34113S-S34114A, S34114D, S34114S-S34115A, S34115D, S34115S, S34119A, S34119D, S34119S, S34121A, S34121D, S34121S-S34122A, S34122D, S34122S-S34123A, S34123D, S34123S-S34124A, S34124D, S34124S-S34125A, S34125D, S34125S, S34129A, S34129D, S34129S, S34131A, S34131D, S34131S-S34132A, S34132D, S34132S, S34139A, S34139D, S34139S, S343XXA |
| Parkinson’s and Huntington’s Disease | ICD-9 codes: 3320-3321, 3330, 3334  ICD-10 codes: G10, G20, G2111, G2119, G212-G214, G218-G219, G230-G232, G238-G239, G903 |
| Pneumonias | ICD-9 codes: 00322, 0064, 0203-0205, 0212, 0221, 0391, 1140, 1144-1145, 11505, 11515, 11595, 1212, 1221, 481, 4820-4822, 48230-48232, 48239-48242, 48249, 48281-48284, 48289, 4847, 5070-5071, 5078, 5100, 5109, 5130-5131, 99731  ICD-10 codes: A0103, A0222, A065, A202, A212, A221, A420, A430, A481, A5484, B380-B382, B390-B392, B400-B402, B410, B664, B671, J13-J14, J150-J151, J1520, J15211-J15212, J1529, J153-J156, J158, J181, J690-J691, J698, J850-J853, J860, J869, J95851 |
| Pressure Ulcer, Skin | ICD-9 codes: 70722-70725  ICD-10 codes: L89000, L89002-L89004, L89010, L89012-L89014, L89020, L89022-L89024, L89100, L89102-L89104, L89110, L89112-L89114, L89120, L89122-L89124, L89130, L89132-L89134, L89140, L89142-L89144, L89150, L89152-L89154, L89200, L89202-L89204, L89210, L89212-L89214, L89220, L89222-L89224, L89300, L89302-L89304, L89310, L89312-L89314, L89320, L89322-L89324, L8942-L8945, L89500, L89502-L89504, L89510, L89512-L89514, L89520, L89522-L89524, L89600, L89602-L89604, L89610, L89612-L89614, L89620, L89622-L89624, L89810, L89812-L89814, L89890, L89892-L89894, L8992-L8995 |
| Protein-Calorie Malnutrition | ICD-9 codes: 260-262, 2630-2632, 2638-2639, 7994  ICD-10 codes: E40-E43, E440-E441, E45-E46, E640, R64 |
| Respirator Dependence or Tracheostomy | ICD-9 codes: 51900-51902, 51909, V440, V4611-V4614, V550  ICD-10 codes: J9500-J9504, J9509, J95850, J95859, Z430, Z930, Z9911-Z9912  ICD-10 codes: J9500-J9504, J9509, J95850, J95859, Z430, Z930, Z9911-Z9912 |
| Rheumatoid Arthritis | ICD-9 codes: 0993, 1361, 4460-4461, 44620-44621, 44629, 4463-4467, 6960, 7100-7105, 7108-7109, 71110-71129, 7140-7142, 71430-71433, 7144, 71481, 71489, 7149, 7200-7202, 72081, 72089, 7209, 725  ICD-10 codes: L4050-L4054, L4059, M0230, M02311-M02312, M02319, M02321-M02322, M02329, M02331-M02332, M02339, M02341-M02342, M02349, M02351-M02352, M02359, M02361-M02362, M02369, M02371-M02372, M02379, M0238-M0239, M041-M042, M048-M049, M0500, M05011-M05012, M05019, M05021-M05022, M05029, M05031-M05032, M05039, M05041-M05042, M05049, M05051-M05052, M05059, M05061-M05062, M05069, M05071-M05072, M05079, M0509-M0510, M05111-M05112, M05119, M05121-M05122, M05129, M05131-M05132, M05139, M05141-M05142, M05149, M05151-M05152, M05159, M05161-M05162, M05169, M05171-M05172, M05179, M0519-M0520, M05211-M05212, M05219, M05221-M05222, M05229, M05231-M05232, M05239, M05241-M05242, M05249, M05251-M05252, M05259, M05261-M05262, M05269, M05271-M05272, M05279, M0529-M0530, M05311-M05312, M05319, M05321-M05322, M05329, M05331-M05332, M05339, M05341-M05342, M05349, M05351-M05352, M05359, M05361-M05362, M05369, M05371-M05372, M05379, M0539-M0540, M05411-M05412, M05419, M05421-M05422, M05429, M05431-M05432, M05439, M05441-M05442, M05449, M05451-M05452, M05459, M05461-M05462, M05469, M05471-M05472, M05479, M0549-M0550, M05511-M05512, M05519, M05521-M05522, M05529, M05531-M05532, M05539, M05541-M05542, M05549, M05551-M05552, M05559, M05561-M05562, M05569, M05571-M05572, M05579, M0559-M0560, M05611-M05612, M05619, M05621-M05622, M05629, M05631-M05632, M05639, M05641-M05642, M05649, M05651-M05652, M05659, M05661-M05662, M05669, M05671-M05672, M05679, M0569-M0570, M05711-M05712, M05719, M05721-M05722, M05729, M05731-M05732, M05739, M05741-M05742, M05749, M05751-M05752, M05759, M05761-M05762, M05769, M05771-M05772, M05779, M0579-M0580, M05811-M05812, M05819, M05821-M05822, M05829, M05831-M05832, M05839, M05841-M05842, M05849, M05851-M05852, M05859, M05861-M05862, M05869, M05871-M05872, M05879, M0589, M059, M0600, M06011-M06012, M06019, M06021-M06022, M06029, M06031-M06032, M06039, M06041-M06042, M06049, M06051-M06052, M06059, M06061-M06062, M06069, M06071-M06072, M06079, M0608-M0609, M061, M0620, M06211-M06212, M06219, M06221-M06222, M06229, M06231-M06232, M06239, M06241-M06242, M06249, M06251-M06252, M06259, M06261-M06262, M06269, M06271-M06272, M06279, M0628-M0630, M06311-M06312, M06319, M06321-M06322, M06329, M06331-M06332, M06339, M06341-M06342, M06349, M06351-M06352, M06359, M06361-M06362, M06369, M06371-M06372, M06379, M0638-M0639, M064, M0680, M06811-M06812, M06819, M06821-M06822, M06829, M06831-M06832, M06839, M06841-M06842, M06849, M06851-M06852, M06859, M06861-M06862, M06869, M06871-M06872, M06879, M0688-M0689, M069, M0800, M08011-M08012, M08019, M08021-M08022, M08029, M08031-M08032, M08039, M08041-M08042, M08049, M08051-M08052, M08059, M08061-M08062, M08069, M08071-M08072, M08079, M0808-M0809, M081, M0820, M08211-M08212, M08219, M08221-M08222, M08229, M08231-M08232, M08239, M08241-M08242, M08249, M08251-M08252, M08259, M08261-M08262, M08269, M08271-M08272, M08279, M0828-M0829, M083, M0840, M08411-M08412, M08419, M08421-M08422, M08429, M08431-M08432, M08439, M08441-M08442, M08449, M08451-M08452, M08459, M08461-M08462, M08469, M08471-M08472, M08479, M0848, M0880, M08811-M08812, M08819, M08821-M08822, M08829, M08831-M08832, M08839, M08841-M08842, M08849, M08851-M08852, M08859, M08861-M08862, M08869, M08871-M08872, M08879, M0888-M0890, M08911-M08912, M08919, M08921-M08922, M08929, M08931-M08932, M08939, M08941-M08942, M08949, M08951-M08952, M08959, M08961-M08962, M08969, M08971-M08972, M08979, M0898-M0899, M1200, M12011-M12012, M12019, M12021-M12022, M12029, M12031-M12032, M12039, M12041-M12042, M12049, M12051-M12052, M12059, M12061-M12062, M12069, M12071-M12072, M12079, M1208-M1209, M300-M303, M308, M310-M312, M3130-M3131, M314-M317, M320, M3210-M3215, M3219, M328-M329, M3300-M3303, M3309-M3313, M3319-M3322, M3329, M3390-M3393, M3399, M340-M342, M3481-M3483, M3489, M349, M3500-M3504, M3509, M351-M353, M355, M358-M360, M368, M450-M459, M4600-M4609, M461, M4650-M4659, M4680-M4699, M488X1-M488X9, M4980-M4989 |
| Schizophrenia and Psychosis | ICD-9 codes: 29500-29505, 29510-29515, 29520-29525, 29530-29535, 29540-29545, 29550-29555, 29560-29565, 29570-29575, 29580-29585, 29590-29595, 2979, 2981, 2983-2984, 2988-2989  ICD-10 codes: F200-F203, F205, F2081, F2089, F209, F23, F250-F251, F258-F259, F28-F29, F531 |
| Seizure and Convulsions | ICD-9 codes: 34500-34501, 34510-34511, 3452-3453, 34540-34541, 34550-34551, 34560-34561, 34570-34571, 34580-34581, 34590-34591, 78031-78033, 78039  ICD-10 codes: G40001, G40009, G40011, G40019, G40101, G40109, G40111, G40119, G40201, G40209, G40211, G40219, G40301, G40309, G40311, G40319, G40401, G40409, G40411, G40419, G40501, G40509, G40801-G40804, G40811-G40814, G40821-G40824, G4089, G40901, G40909, G40911, G40919, G40A01, G40A09, G40A11, G40A19, G40B01, G40B09, G40B11, G40B19, R5600-R5601, R561, R569 |
| Sepsis/Shock | ICD-9 codes: 0031, 0202, 0223, 0362, 0380, 03810-03812, 03819, 0382-0383, 03840-03844, 03849, 0388-0389, 04082, 0545, 77181, 78552, 78559, 99590-99594, 99802  ICD-10 codes: A021, A207, A227, A267, A327, A392-A394, A400-A401, A403, A408-A409, A4101-A4102, A411-A414, A4150-A4153, A4159, A4181, A4189, A419, A427, A483, A5486, B007, B377, P0270, P360, P3610, P3619, P362, P3630, P3639, P364-P365, P368-P369, R571, R578, R6510-R6511, R6520-R6521, T8112XA, T8144XA |
| Substance Abuse with Complications | ICD-9 codes: 2910-2915, 29181-29182, 29189, 2919-2920, 29211-29212, 2922, 29281-29285, 29289, 2929, 30300-30303, 30390-30393, 30400-30403, 30410-30413, 30420-30423, 30430-30433, 30440-30443, 30450-30453, 30460-30463, 30470-30473, 30480-30483, 30490-30493, 96500-96502, 96509, 9696, 96970-96973, 96979, 97081, 9800, E8500-E8502, E8541-E8542, E8601, E9800, E9802-E9804, E9809  ICD-10 codes: F10120-F10121, F10129, F1014, F10150-F10151, F10159, F10180-F10182, F10188, F1019-F1021, F10220-F10221, F10229-F10232, F10239, F1024, F10250-F10251, F10259, F1026-F1027, F10280-F10282, F10288, F1029, F10920-F10921, F10929, F1094, F10950-F10951, F10959, F1096-F1097, F10980-F10982, F10988, F1099, F11120-F11122, F11129, F1114, F11150-F11151, F11159, F11181-F11182, F11188, F1119-F1121, F11220-F11222, F11229, F1123-F1124, F11250-F11251, F11259, F11281-F11282, F11288, F1129, F11920-F11922, F11929, F1193-F1194, F11950-F11951, F11959, F11981-F11982, F11988, F1199, F12120-F12122, F12129, F12150-F12151, F12159, F12180, F12188, F1219-F1221, F12220-F12222, F12229, F1223, F12250-F12251, F12259, F12280, F12288, F1229, F12920-F12922, F12929, F1293, F12950-F12951, F12959, F12980, F12988, F1299, F13120-F13121, F13129, F1314, F13150-F13151, F13159, F13180-F13182, F13188, F1319-F1321, F13220-F13221, F13229-F13232, F13239, F1324, F13250-F13251, F13259, F1326-F1327, F13280-F13282, F13288, F1329, F13920-F13921, F13929-F13932, F13939, F1394, F13950-F13951, F13959, F1396-F1397, F13980-F13982, F13988, F1399, F14120-F14122, F14129, F1414, F14150-F14151, F14159, F14180-F14182, F14188, F1419-F1421, F14220-F14222, F14229, F1423-F1424, F14250-F14251, F14259, F14280-F14282, F14288, F1429, F14920-F14922, F14929, F1494, F14950-F14951, F14959, F14980-F14982, F14988, F1499, F15120-F15122, F15129, F1514, F15150-F15151, F15159, F15180-F15182, F15188, F1519-F1521, F15220-F15222, F15229, F1523-F1524, F15250-F15251, F15259, F15280-F15282, F15288, F1529, F15920-F15922, F15929, F1593-F1594, F15950-F15951, F15959, F15980-F15982, F15988, F1599, F16120-F16122, F16129, F1614, F16150-F16151, F16159, F16180, F16183, F16188, F1619-F1621, F16220-F16221, F16229, F1624, F16250-F16251, F16259, F16280, F16283, F16288, F1629, F16920-F16921, F16929, F1694, F16950-F16951, F16959, F16980, F16983, F16988, F1699, F18120-F18121, F18129, F1814, F18150-F18151, F18159, F1817, F18180, F18188, F1819-F1821, F18220-F18221, F18229, F1824, F18250-F18251, F18259, F1827, F18280, F18288, F1829, F18920-F18921, F18929, F1894, F18950-F18951, F18959, F1897, F18980, F18988, F1899, F19120-F19122, F19129, F1914, F19150-F19151, F19159, F1916-F1917, F19180-F19182, F19188, F1919-F1921, F19220-F19222, F19229-F19232, F19239, F1924, F19250-F19251, F19259, F1926-F1927, F19280-F19282, F19288, F1929, F19920-F19922, F19929-F19932, F19939, F1994, F19950-F19951, F19959, F1996-F1997, F19980-F19982, F19988, F1999, T400X1A, T400X4A, T401X1A, T401X4A, T402X1A, T402X4A, T403X1A, T403X4A, T404X1A, T404X4A, T405X1A, T405X4A, T40601A, T40604A, T40691A, T40694A, T408X1A, T408X4A, T40901A, T40904A, T40991A, T40994A, T43601A, T43604A, T43611A, T43614A, T43621A, T43624A, T43631A, T43634A, T43641A, T43644A, T43691A, T43694A, T510X1A, T510X4A |
| Thyroid Disorders | ICD-9 codes: 2400, 2409-2411, 2419, 24200-24201, 24210-24211, 24220-24221, 24230-24231, 24240-24241, 24280-24281, 24290-24291, 243, 2440-2443, 2448-2454, 2458-2463, 2468-2469  ICD-10 codes: E000-E002, E009-E012, E018, E02, E030-E035, E038-E042, E048-E049, E0500-E0501, E0510-E0511, E0520-E0521, E0530-E0531, E0540-E0541, E0580-E0581, E0590-E0591, E060-E065, E069-E071, E0789, E079, E890 |
| Trauma, Head | ICD-9 codes: 80000-80006, 80009-80016, 80019-80026, 80029-80036, 80039-80046, 80049-80056, 80059-80066, 80069-80076, 80079-80086, 80089-80096, 80099-80106, 80109-80116, 80119-80126, 80129-80136, 80139-80146, 80149-80156, 80159-80166, 80169-80176, 80179-80186, 80189-80196, 80199, 80220-80239, 8024-8029, 80300-80306, 80309-80316, 80319-80326, 80329-80336, 80339-80346, 80349-80356, 80359-80366, 80369-80376, 80379-80386, 80389-80396, 80399-80406, 80409-80416, 80419-80426, 80429-80436, 80439-80446, 80449-80456, 80459-80466, 80469-80476, 80479-80486, 80489-80496, 80499, 8502-8504, 85100-85106, 85109-85116, 85119-85126, 85129-85136, 85139-85146, 85149-85156, 85159-85166, 85169-85176, 85179-85186, 85189-85196, 85199-85206, 85209-85216, 85219-85226, 85229-85236, 85239-85246, 85249-85256, 85259, 85300-85306, 85309-85316, 85319, 85400-85406, 85409-85416, 85419, 9050, 9070  ICD-10 codes: S020XXA, S020XXB, S020XXS, S02101A, S02101B, S02101S-S02102A, S02102B, S02102S, S02109A, S02109B, S02109S-S02110A, S02110B, S02110S-S02111A, S02111B, S02111S-S02112A, S02112B, S02112S-S02113A, S02113B, S02113S, S02118A, S02118B, S02118S-S02119A, S02119B, S02119S, S0211AA, S0211AB, S0211AS, S0211BA, S0211BB, S0211BS, S0211CA, S0211CB, S0211CS, S0211DA, S0211DB, S0211DS, S0211EA, S0211EB, S0211ES, S0211FA, S0211FB, S0211FS, S0211GA, S0211GB, S0211GS, S0211HA, S0211HB, S0211HS, S02121A, S02121B, S02121S-S02122A, S02122B, S02122S, S02129A, S02129B, S02129S, S0219XA, S0219XB, S0219XS, S0230XA, S0230XB, S0230XS-S0231XA, S0231XB, S0231XS-S0232XA, S0232XB, S0232XS, S02400A, S02400B, S02400S-S02401A, S02401B, S02401S-S02402A, S02402B, S02402S, S0240AA, S0240AB, S0240AS, S0240BA, S0240BB, S0240BS, S0240CA, S0240CB, S0240CS, S0240DA, S0240DB, S0240DS, S0240EA, S0240EB, S0240ES, S0240FA, S0240FB, S0240FS, S02411A, S02411B, S02411S-S02412A, S02412B, S02412S-S02413A, S02413B, S02413S, S0242XA, S0242XB, S0242XS, S02600A, S02600B, S02600S-S02601A, S02601B, S02601S-S02602A, S02602B, S02602S, S02609A, S02609B, S02609S-S02610A, S02610B, S02610S-S02611A, S02611B, S02611S-S02612A, S02612B, S02612S, S02620A, S02620B, S02620S-S02621A, S02621B, S02621S-S02622A, S02622B, S02622S, S02630A, S02630B, S02630S-S02631A, S02631B, S02631S-S02632A, S02632B, S02632S, S02640A, S02640B, S02640S-S02641A, S02641B, S02641S-S02642A, S02642B, S02642S, S02650A, S02650B, S02650S-S02651A, S02651B, S02651S-S02652A, S02652B, S02652S, S0266XA, S0266XB, S0266XS, S02670A, S02670B, S02670S-S02671A, S02671B, S02671S-S02672A, S02672B, S02672S, S0269XA, S0269XB, S0269XS, S0280XA, S0280XB, S0280XS-S0281XA, S0281XB, S0281XS-S0282XA, S0282XB, S0282XS, S02831A, S02831B, S02831S-S02832A, S02832B, S02832S, S02839A, S02839B, S02839S, S02841A, S02841B, S02841S-S02842A, S02842B, S02842S, S02849A, S02849B, S02849S, S0285XA, S0285XB, S0285XS, S0291XA, S0291XB, S0291XS-S0292XA, S0292XB, S0292XS, S060X0S-S060X1S, S060X9S-S061X0A, S061X0S-S061X1A, S061X1S-S061X2A, S061X2S-S061X3A, S061X3S-S061X4A, S061X4S-S061X5A, S061X5S-S061X6A, S061X6S, S061X9A, S061X9S-S062X0A, S062X0S-S062X1A, S062X1S-S062X2A, S062X2S-S062X3A, S062X3S-S062X4A, S062X4S-S062X5A, S062X5S-S062X6A, S062X6S, S062X9A, S062X9S, S06300A, S06300S-S06301A, S06301S-S06302A, S06302S-S06303A, S06303S-S06304A, S06304S-S06305A, S06305S-S06306A, S06306S, S06309A, S06309S-S06310A, S06310S-S06311A, S06311S-S06312A, S06312S-S06313A, S06313S-S06314A, S06314S-S06315A, S06315S-S06316A, S06316S, S06319A, S06319S-S06320A, S06320S-S06321A, S06321S-S06322A, S06322S-S06323A, S06323S-S06324A, S06324S-S06325A, S06325S-S06326A, S06326S, S06329A, S06329S-S06330A, S06330S-S06331A, S06331S-S06332A, S06332S-S06333A, S06333S-S06334A, S06334S-S06335A, S06335S-S06336A, S06336S, S06339A, S06339S-S06340A, S06340S-S06341A, S06341S-S06342A, S06342S-S06343A, S06343S-S06344A, S06344S-S06345A, S06345S-S06346A, S06346S, S06349A, S06349S-S06350A, S06350S-S06351A, S06351S-S06352A, S06352S-S06353A, S06353S-S06354A, S06354S-S06355A, S06355S-S06356A, S06356S, S06359A, S06359S-S06360A, S06360S-S06361A, S06361S-S06362A, S06362S-S06363A, S06363S-S06364A, S06364S-S06365A, S06365S-S06366A, S06366S, S06369A, S06369S-S06370A, S06370S-S06371A, S06371S-S06372A, S06372S-S06373A, S06373S-S06374A, S06374S-S06375A, S06375S-S06376A, S06376S, S06379A, S06379S-S06380A, S06380S-S06381A, S06381S-S06382A, S06382S-S06383A, S06383S-S06384A, S06384S-S06385A, S06385S-S06386A, S06386S, S06389A, S06389S, S064X0A, S064X0S-S064X1A, S064X1S-S064X2A, S064X2S-S064X3A, S064X3S-S064X4A, S064X4S-S064X5A, S064X5S-S064X6A, S064X6S, S064X9A, S064X9S-S065X0A, S065X0S-S065X1A, S065X1S-S065X2A, S065X2S-S065X3A, S065X3S-S065X4A, S065X4S-S065X5A, S065X5S-S065X6A, S065X6S, S065X9A, S065X9S-S066X0A, S066X0S-S066X1A, S066X1S-S066X2A, S066X2S-S066X3A, S066X3S-S066X4A, S066X4S-S066X5A, S066X5S-S066X6A, S066X6S, S066X9A, S066X9S, S06810A, S06810S-S06811A, S06811S-S06812A, S06812S-S06813A, S06813S-S06814A, S06814S-S06815A, S06815S-S06816A, S06816S, S06819A, S06819S-S06820A, S06820S-S06821A, S06821S-S06822A, S06822S-S06823A, S06823S-S06824A, S06824S-S06825A, S06825S-S06826A, S06826S, S06829A, S06829S, S06890A, S06890S-S06891A, S06891S-S06892A, S06892S-S06893A, S06893S-S06894A, S06894S-S06895A, S06895S-S06896A, S06896S, S06899A, S06899S, S069X0A, S069X0S-S069X1A, S069X1S-S069X2A, S069X2S-S069X3A, S069X3S-S069X4A, S069X4S-S069X5A, S069X5S-S069X6A, S069X6S, S069X9A, S069X9S |
| Trauma, Other | ICD-9 codes: 73313-73315, 80500-80508, 80510-80518, 8052-8059, 8080-8083, 80841-80844, 80849, 80851-80854, 80859, 8088-8089, 82000-82003, 82009-82013, 82019-82022, 82030-82032, 8208-8209, 82100-82101, 82110-82111, 82120-82123, 82129-82133, 82139, 83500-83503, 83510-83513, 8870-8877, 8950-8951, 8960-8963, 8970-8977, 9580-9588, 95890-95893, 95899, 99690-99696, 99699  ICD-10 codes: M4850XA-M4858XA, M80051A-M80052A, M80059A, M8008XA, M80851A-M80852A, M80859A, M8088XA, M84451A-M84453A, M84459A, M84551A-M84553A, M84559A, M84651A-M84653A, M84659A, M84754A-M84759A, M9701XA-M9702XA, S12000A, S12000B-S12001A, S12001B, S1201XA, S1201XB-S1202XA, S1202XB, S12030A, S12030B-S12031A, S12031B, S12040A, S12040B-S12041A, S12041B, S12090A, S12090B-S12091A, S12091B, S12100A, S12100B-S12101A, S12101B, S12110A, S12110B-S12111A, S12111B-S12112A, S12112B, S12120A, S12120B-S12121A, S12121B, S12130A, S12130B-S12131A, S12131B, S1214XA, S1214XB, S12150A, S12150B-S12151A, S12151B, S12190A, S12190B-S12191A, S12191B, S12200A, S12200B-S12201A, S12201B, S12230A, S12230B-S12231A, S12231B, S1224XA, S1224XB, S12250A, S12250B-S12251A, S12251B, S12290A, S12290B-S12291A, S12291B, S12300A, S12300B-S12301A, S12301B, S12330A, S12330B-S12331A, S12331B, S1234XA, S1234XB, S12350A, S12350B-S12351A, S12351B, S12390A, S12390B-S12391A, S12391B, S12400A, S12400B-S12401A, S12401B, S12430A, S12430B-S12431A, S12431B, S1244XA, S1244XB, S12450A, S12450B-S12451A, S12451B, S12490A, S12490B-S12491A, S12491B, S12500A, S12500B-S12501A, S12501B, S12530A, S12530B-S12531A, S12531B, S1254XA, S1254XB, S12550A, S12550B-S12551A, S12551B, S12590A, S12590B-S12591A, S12591B, S12600A, S12600B-S12601A, S12601B, S12630A, S12630B-S12631A, S12631B, S1264XA, S1264XB, S12650A, S12650B-S12651A, S12651B, S12690A, S12690B-S12691A, S12691B, S128XXA-S129XXA, S22000A, S22000B-S22001A, S22001B-S22002A, S22002B, S22008A, S22008B-S22009A, S22009B-S22010A, S22010B-S22011A, S22011B-S22012A, S22012B, S22018A, S22018B-S22019A, S22019B-S22020A, S22020B-S22021A, S22021B-S22022A, S22022B, S22028A, S22028B-S22029A, S22029B-S22030A, S22030B-S22031A, S22031B-S22032A, S22032B, S22038A, S22038B-S22039A, S22039B-S22040A, S22040B-S22041A, S22041B-S22042A, S22042B, S22048A, S22048B-S22049A, S22049B-S22050A, S22050B-S22051A, S22051B-S22052A, S22052B, S22058A, S22058B-S22059A, S22059B-S22060A, S22060B-S22061A, S22061B-S22062A, S22062B, S22068A, S22068B-S22069A, S22069B-S22070A, S22070B-S22071A, S22071B-S22072A, S22072B, S22078A, S22078B-S22079A, S22079B-S22080A, S22080B-S22081A, S22081B-S22082A, S22082B, S22088A, S22088B-S22089A, S22089B, S32000A, S32000B-S32001A, S32001B-S32002A, S32002B, S32008A, S32008B-S32009A, S32009B-S32010A, S32010B-S32011A, S32011B-S32012A, S32012B, S32018A, S32018B-S32019A, S32019B-S32020A, S32020B-S32021A, S32021B-S32022A, S32022B, S32028A, S32028B-S32029A, S32029B-S32030A, S32030B-S32031A, S32031B-S32032A, S32032B, S32038A, S32038B-S32039A, S32039B-S32040A, S32040B-S32041A, S32041B-S32042A, S32042B, S32048A, S32048B-S32049A, S32049B-S32050A, S32050B-S32051A, S32051B-S32052A, S32052B, S32058A, S32058B-S32059A, S32059B, S3210XA, S3210XB, S32110A, S32110B-S32111A, S32111B-S32112A, S32112B, S32119A, S32119B-S32120A, S32120B-S32121A, S32121B-S32122A, S32122B, S32129A, S32129B-S32130A, S32130B-S32131A, S32131B-S32132A, S32132B, S32139A, S32139B, S3214XA, S3214XB-S3215XA, S3215XB-S3216XA, S3216XB-S3217XA, S3217XB, S3219XA, S3219XB, S322XXA, S322XXB, S32301A, S32301B-S32302A, S32302B, S32309A, S32309B, S32311A, S32311B-S32312A, S32312B-S32313A, S32313B-S32314A, S32314B-S32315A, S32315B-S32316A, S32316B, S32391A, S32391B-S32392A, S32392B, S32399A, S32399B, S32401A, S32401B-S32402A, S32402B, S32409A, S32409B, S32411A, S32411B-S32412A, S32412B-S32413A, S32413B-S32414A, S32414B-S32415A, S32415B-S32416A, S32416B, S32421A, S32421B-S32422A, S32422B-S32423A, S32423B-S32424A, S32424B-S32425A, S32425B-S32426A, S32426B, S32431A, S32431B-S32432A, S32432B-S32433A, S32433B-S32434A, S32434B-S32435A, S32435B-S32436A, S32436B, S32441A, S32441B-S32442A, S32442B-S32443A, S32443B-S32444A, S32444B-S32445A, S32445B-S32446A, S32446B, S32451A, S32451B-S32452A, S32452B-S32453A, S32453B-S32454A, S32454B-S32455A, S32455B-S32456A, S32456B, S32461A, S32461B-S32462A, S32462B-S32463A, S32463B-S32464A, S32464B-S32465A, S32465B-S32466A, S32466B, S32471A, S32471B-S32472A, S32472B-S32473A, S32473B-S32474A, S32474B-S32475A, S32475B-S32476A, S32476B, S32481A, S32481B-S32482A, S32482B-S32483A, S32483B-S32484A, S32484B-S32485A, S32485B-S32486A, S32486B, S32491A, S32491B-S32492A, S32492B, S32499A, S32499B, S32501A, S32501B-S32502A, S32502B, S32509A, S32509B, S32511A, S32511B-S32512A, S32512B, S32519A, S32519B, S32591A, S32591B-S32592A, S32592B, S32599A, S32599B, S32601A, S32601B-S32602A, S32602B, S32609A, S32609B, S32611A, S32611B-S32612A, S32612B-S32613A, S32613B-S32614A, S32614B-S32615A, S32615B-S32616A, S32616B, S32691A, S32691B-S32692A, S32692B, S32699A, S32699B, S32810A, S32810B-S32811A, S32811B, S3282XA, S3282XB, S3289XA, S3289XB, S329XXA, S329XXB, S48011A-S48012A, S48019A, S48021A-S48022A, S48029A, S48111A-S48112A, S48119A, S48121A-S48122A, S48129A, S48911A-S48912A, S48919A, S48921A-S48922A, S48929A, S58011A-S58012A, S58019A, S58021A-S58022A, S58029A, S58111A-S58112A, S58119A, S58121A-S58122A, S58129A, S58911A-S58912A, S58919A, S58921A-S58922A, S58929A, S68411A-S68412A, S68419A, S68421A-S68422A, S68429A, S68711A-S68712A, S68719A, S68721A-S68722A, S68729A, S72001A, S72001B, S72001C-S72002A, S72002B, S72002C, S72009A, S72009B, S72009C, S72011A, S72011B, S72011C-S72012A, S72012B, S72012C, S72019A, S72019B, S72019C, S72021A, S72021B, S72021C-S72022A, S72022B, S72022C-S72023A, S72023B, S72023C-S72024A, S72024B, S72024C-S72025A, S72025B, S72025C-S72026A, S72026B, S72026C, S72031A, S72031B, S72031C-S72032A, S72032B, S72032C-S72033A, S72033B, S72033C-S72034A, S72034B, S72034C-S72035A, S72035B, S72035C-S72036A, S72036B, S72036C, S72041A, S72041B, S72041C-S72042A, S72042B, S72042C-S72043A, S72043B, S72043C-S72044A, S72044B, S72044C-S72045A, S72045B, S72045C-S72046A, S72046B, S72046C, S72051A, S72051B, S72051C-S72052A, S72052B, S72052C, S72059A, S72059B, S72059C, S72061A, S72061B, S72061C-S72062A, S72062B, S72062C-S72063A, S72063B, S72063C-S72064A, S72064B, S72064C-S72065A, S72065B, S72065C-S72066A, S72066B, S72066C, S72091A, S72091B, S72091C-S72092A, S72092B, S72092C, S72099A, S72099B, S72099C, S72101A, S72101B, S72101C-S72102A, S72102B, S72102C, S72109A, S72109B, S72109C, S72111A, S72111B, S72111C-S72112A, S72112B, S72112C-S72113A, S72113B, S72113C-S72114A, S72114B, S72114C-S72115A, S72115B, S72115C-S72116A, S72116B, S72116C, S72121A, S72121B, S72121C-S72122A, S72122B, S72122C-S72123A, S72123B, S72123C-S72124A, S72124B, S72124C-S72125A, S72125B, S72125C-S72126A, S72126B, S72126C, S72131A, S72131B, S72131C-S72132A, S72132B, S72132C-S72133A, S72133B, S72133C-S72134A, S72134B, S72134C-S72135A, S72135B, S72135C-S72136A, S72136B, S72136C, S72141A, S72141B, S72141C-S72142A, S72142B, S72142C-S72143A, S72143B, S72143C-S72144A, S72144B, S72144C-S72145A, S72145B, S72145C-S72146A, S72146B, S72146C, S7221XA, S7221XB, S7221XC-S7222XA, S7222XB, S7222XC-S7223XA, S7223XB, S7223XC-S7224XA, S7224XB, S7224XC-S7225XA, S7225XB, S7225XC-S7226XA, S7226XB, S7226XC, S72301A, S72301B, S72301C-S72302A, S72302B, S72302C, S72309A, S72309B, S72309C, S72321A, S72321B, S72321C-S72322A, S72322B, S72322C-S72323A, S72323B, S72323C-S72324A, S72324B, S72324C-S72325A, S72325B, S72325C-S72326A, S72326B, S72326C, S72331A, S72331B, S72331C-S72332A, S72332B, S72332C-S72333A, S72333B, S72333C-S72334A, S72334B, S72334C-S72335A, S72335B, S72335C-S72336A, S72336B, S72336C, S72341A, S72341B, S72341C-S72342A, S72342B, S72342C-S72343A, S72343B, S72343C-S72344A, S72344B, S72344C-S72345A, S72345B, S72345C-S72346A, S72346B, S72346C, S72351A, S72351B, S72351C-S72352A, S72352B, S72352C-S72353A, S72353B, S72353C-S72354A, S72354B, S72354C-S72355A, S72355B, S72355C-S72356A, S72356B, S72356C, S72361A, S72361B, S72361C-S72362A, S72362B, S72362C-S72363A, S72363B, S72363C-S72364A, S72364B, S72364C-S72365A, S72365B, S72365C-S72366A, S72366B, S72366C, S72391A, S72391B, S72391C-S72392A, S72392B, S72392C, S72399A, S72399B, S72399C, S72401A, S72401B, S72401C-S72402A, S72402B, S72402C, S72409A, S72409B, S72409C, S72411A, S72411B, S72411C-S72412A, S72412B, S72412C-S72413A, S72413B, S72413C-S72414A, S72414B, S72414C-S72415A, S72415B, S72415C-S72416A, S72416B, S72416C, S72421A, S72421B, S72421C-S72422A, S72422B, S72422C-S72423A, S72423B, S72423C-S72424A, S72424B, S72424C-S72425A, S72425B, S72425C-S72426A, S72426B, S72426C, S72431A, S72431B, S72431C-S72432A, S72432B, S72432C-S72433A, S72433B, S72433C-S72434A, S72434B, S72434C-S72435A, S72435B, S72435C-S72436A, S72436B, S72436C, S72441A, S72441B, S72441C-S72442A, S72442B, S72442C-S72443A, S72443B, S72443C-S72444A, S72444B, S72444C-S72445A, S72445B, S72445C-S72446A, S72446B, S72446C, S72451A, S72451B, S72451C-S72452A, S72452B, S72452C-S72453A, S72453B, S72453C-S72454A, S72454B, S72454C-S72455A, S72455B, S72455C-S72456A, S72456B, S72456C, S72461A, S72461B, S72461C-S72462A, S72462B, S72462C-S72463A, S72463B, S72463C-S72464A, S72464B, S72464C-S72465A, S72465B, S72465C-S72466A, S72466B, S72466C, S72471A-S72472A, S72479A, S72491A, S72491B, S72491C-S72492A, S72492B, S72492C, S72499A, S72499B, S72499C, S728X1A, S728X1B, S728X1C-S728X2A, S728X2B, S728X2C, S728X9A, S728X9B, S728X9C-S7290XA, S7290XB, S7290XC-S7291XA, S7291XB, S7291XC-S7292XA, S7292XB, S7292XC, S73001A-S73006A, S73011A-S73016A, S73021A-S73026A, S73031A-S73036A, S73041A-S73046A, S78011A-S78012A, S78019A, S78021A-S78022A, S78029A, S78111A-S78112A, S78119A, S78121A-S78122A, S78129A, S78911A-S78912A, S78919A, S78921A-S78922A, S78929A, S79001A-S79002A, S79009A, S79011A-S79012A, S79019A, S79091A-S79092A, S79099A, S79101A-S79102A, S79109A, S79111A-S79112A, S79119A, S79121A-S79122A, S79129A, S79131A-S79132A, S79139A, S79141A-S79142A, S79149A, S79191A-S79192A, S79199A, S88011A-S88012A, S88019A, S88021A-S88022A, S88029A, S88111A-S88112A, S88119A, S88121A-S88122A, S88129A, S88911A-S88912A, S88919A, S88921A-S88922A, S88929A, S98011A-S98012A, S98019A, S98021A-S98022A, S98029A, S98111A-S98112A, S98119A, S98121A-S98122A, S98129A, S98131A-S98132A, S98139A, S98141A-S98142A, S98149A, S98211A-S98212A, S98219A, S98221A-S98222A, S98229A, S98311A-S98312A, S98319A, S98321A-S98322A, S98329A, S98911A-S98912A, S98919A, S98921A-S98922A, S98929A, T790XXA-T792XXA, T794XXA-T799XXA, T79A0XA, T79A11A-T79A12A, T79A19A, T79A21A-T79A22A, T79A29A, T79A3XA, T79A9XA, T870X1-T870X2, T870X9, T871X1-T871X2, T871X9, T872 |
| Unstable Angina and Other Ischemic Heart Disease | ICD-9 codes: 41000, 41002, 41010, 41012, 41020, 41022, 41030, 41032, 41040, 41042, 41050, 41052, 41060, 41062, 41070, 41072, 41080, 41082, 41090, 41092, 4110-4111, 41181, 41189  ICD-10 codes: I200, I230-I233, I236-I238, I240-I241, I248-I249, I25110, I25700, I25710, I25720, I25730, I25750, I25760, I25790 |
| Vascular Diseases | ICD-9 codes: 0400, 41511-41513, 41519, 4162, 4400-4401, 44020-44024, 44029-44032, 4404, 44100-44103, 4411-4417, 4419-4423, 44281-44284, 44289, 4429, 4431, 44321-44324, 44329, 44381-44382, 44389, 4439, 44401, 44409, 4441, 44421-44422, 44481, 44489, 4449, 44501-44502, 44581, 44589, 4470-4476, 44770-44773, 4478-4480, 449, 45111, 45119, 45181, 45183, 4530, 4532-4533, 45340-45342, 45350-45352, 45372, 45374-45377, 45382, 45384-45387, 4540, 4542, 45911, 45913, 45931, 45933, 5570-5571, 5579, 59381, 7854  ICD-10 codes: A480, E0851-E0852, E0852, E0951-E0952, E0952, E1051-E1052, E1052, E1151-E1152, E1152, E1351-E1352, E1352, I2601-I2602, I2609, I2690, I2692-I2694, I2699, I2782, I670, I700-I701, I70201-I70203, I70208-I70209, I70211-I70213, I70218-I70219, I70221-I70223, I70228-I70229, I70231-I70235, I70238-I70239, I70241-I70245, I70248-I70249, I7025, I70261-I70263, I70268-I70269, I70291-I70293, I70298-I70299, I70301-I70303, I70308-I70309, I70311-I70313, I70318-I70319, I70321-I70323, I70328-I70329, I70331-I70335, I70338-I70339, I70341-I70345, I70348-I70349, I7035, I70361-I70363, I70368-I70369, I70391-I70393, I70398-I70399, I70401-I70403, I70408-I70409, I70411-I70413, I70418-I70419, I70421-I70423, I70428-I70429, I70431-I70435, I70438-I70439, I70441-I70445, I70448-I70449, I7045, I70461-I70463, I70468-I70469, I70491-I70493, I70498-I70499, I70501-I70503, I70508-I70509, I70511-I70513, I70518-I70519, I70521-I70523, I70528-I70529, I70531-I70535, I70538-I70539, I70541-I70545, I70548-I70549, I7055, I70561-I70563, I70568-I70569, I70591-I70593, I70598-I70599, I70601-I70603, I70608-I70609, I70611-I70613, I70618-I70619, I70621-I70623, I70628-I70629, I70631-I70635, I70638-I70639, I70641-I70645, I70648-I70649, I7065, I70661-I70663, I70668-I70669, I70691-I70693, I70698-I70699, I70701-I70703, I70708-I70709, I70711-I70713, I70718-I70719, I70721-I70723, I70728-I70729, I70731-I70735, I70738-I70739, I70741-I70745, I70748-I70749, I7075, I70761-I70763, I70768-I70769, I70791-I70793, I70798-I70799, I7092, I7100-I7103, I711-I716, I718-I726, I728-I729, I7301, I731, I7381, I7389, I739, I7401, I7409-I7411, I7419, I742-I745, I748-I749, I75011-I75013, I75019, I75021-I75023, I75029, I7581, I7589, I76, I770-I776, I7770-I7777, I7779, I77810-I77812, I77819, I7789, I779-I780, I790-I791, I798, I8010-I8013, I80201-I80203, I80209, I80211-I80213, I80219, I80221-I80223, I80229, I80231-I80233, I80239, I80241-I80243, I80249, I80251-I80253, I80259, I80291-I80293, I80299, I820, I82210-I82211, I82220-I82221, I82290-I82291, I823, I82401-I82403, I82409, I82411-I82413, I82419, I82421-I82423, I82429, I82431-I82433, I82439, I82441-I82443, I82449, I82451-I82453, I82459, I82461-I82463, I82469, I82491-I82493, I82499, I824Y1-I824Y3, I824Y9, I824Z1-I824Z3, I824Z9, I82501-I82503, I82509, I82511-I82513, I82519, I82521-I82523, I82529, I82531-I82533, I82539, I82541-I82543, I82549, I82551-I82553, I82559, I82561-I82563, I82569, I82591-I82593, I82599, I825Y1-I825Y3, I825Y9, I825Z1-I825Z3, I825Z9, I82621-I82623, I82629, I82721-I82723, I82729, I82A11-I82A13, I82A19, I82A21-I82A23, I82A29, I82B11-I82B13, I82B19, I82B21-I82B23, I82B29, I82C11-I82C13, I82C19, I82C21-I82C23, I82C29, I83001-I83005, I83008-I83009, I83011-I83015, I83018-I83019, I83021-I83025, I83028-I83029, I83201-I83205, I83208-I83209, I83211-I83215, I83218-I83219, I83221-I83225, I83228-I83229, I87011-I87013, I87019, I87031-I87033, I87039, I87311-I87313, I87319, I87331-I87333, I87339, I96, K55011-K55012, K55019, K55021-K55022, K55029, K55031-K55032, K55039, K55041-K55042, K55049, K55051-K55052, K55059, K55061-K55062, K55069, K551, K5530-K5533, K558-K559, M318-M319, N280 |
| Wheelchair/Beds | CPT/HCPCS codes: E0971, E0973, E1050, E1060, E1070, E1083-E1090, E1092-E1093, E1100, E1110, E1140, E1150, E1160-E1161, E1170, E1250, E1260, E1285, E1290, E2365, E2601, K0001-K0014, K0195, K0813-K0816, K0820-K0831, K0835-K0843, K0848-K0864, K0868-K0871, K0877-K0880, K0884-K0886, K0890-K0891, K0898, A0130, E0250-E0251, E0255-E0256, E0260-E0261, E0265-E0266, E0270, E0290-E0297, E0301-E0304, E0316 |

HIV/AIDS = Human Immunodeficiency Virus/Acquired Immunodeficiency Syndrome

**Table S2.** Silber Qualifying Comorbidity Sets Comprised of Single, Double, and Triple Combinations.

| **Single Condition Qualifying Comorbidity Sets** | | |
| --- | --- | --- |
| Acute Myocardial Infarction |  |  |
| Amputation and Complications |  |  |
| CKD Stage 4-5 and Dialysis |  |  |
| Cardio and Respiratory Failure |  |  |
| Oxygen |  |  |
| Liver Diseases |  |  |
| Pneumonia |  |  |
| Pressure Ulcer, Skin |  |  |
| Protein-Calorie Malnutrition |  |  |
| Sepsis/Shock |  |  |
| **Double Condition Qualifying Comorbidity Sets** | | |
| Acute Renal Failure | Wheelchair/Beds |  |
| Acute Renal Failure | Chronic Ulcer, Skin, Not Pressure |  |
| Acute Renal Failure | Other Trauma |  |
| Congestive Heart Failure | Wheelchair/Beds |  |
| Congestive Heart Failure | Acute Renal Failure |  |
| Congestive Heart Failure | Chronic Lung Diseases |  |
| Congestive Heart Failure | Chronic Ulcer, Skin, Not Pressure |  |
| Congestive Heart Failure | Complications Implants Graft |  |
| Congestive Heart Failure | Other Trauma |  |
| Congestive Heart Failure | Vascular Diseases |  |
| Chronic Lung Diseases | Wheelchair/Beds |  |
| Chronic Lung Diseases | Acute Renal Failure |  |
| Chronic Lung Diseases | Chronic Ulcer, Skin, Not Pressure |  |
| Chronic Lung Diseases | Other Trauma |  |
| Chronic Ulcer, Skin, Not Pressure | Complications Implants Graft |  |
| Chronic Ulcer, Skin, Not Pressure | Coronary Artery Disease |  |
| Diabetes with Complications | Congestive Heart Failure |  |
| Diabetes with Complications | Chronic Ulcer, Skin, Not Pressure |  |
| Diabetes with Complications | Complications Implants Graft |  |
| Diabetes with Complications | Other Hematological |  |
| Diabetes with Complications | Other Trauma |  |
| Endocrine and Metabolic Disorders | Chronic Ulcer, Skin, Not Pressure |  |
| Endocrine and Metabolic Disorders | Other Hematological |  |
| Heart Arrhythmias | Acute Renal Failure |  |
| Heart Arrhythmias | Chronic Lung Diseases |  |
| Heart Arrhythmias | Chronic Ulcer, Skin, Not Pressure |  |
| Heart Arrhythmias | Other Trauma |  |
| Other Hematological | Wheelchair/Beds |  |
| Other Hematological | Acute Renal Failure |  |
| Other Hematological | Congestive Heart Failure |  |
| Other Hematological | Chronic Lung Diseases |  |
| Other Hematological | Chronic Ulcer, Skin, Not Pressure |  |
| Other Hematological | Complications Implants Graft |  |
| Other Hematological | Coronary Artery Disease |  |
| Other Hematological | Other Depression |  |
| Other Hematological | Heart Arrhythmias |  |
| Other Hematological | Hypertension |  |
| Other Hematological | Substance Abuse with Complications |  |
| Other Hematological | Other Trauma |  |
| Other Hematological | Vascular Diseases |  |
| Disorders of Immunity | Acute Renal Failure |  |
| Opportunistic Infections | Vascular Diseases |  |
| Vascular Diseases | Wheelchair/Beds |  |
| Vascular Diseases | Acute Renal Failure |  |
| Vascular Diseases | Chronic Ulcer, Skin, Not Pressure |  |
| Vascular Diseases | Other Trauma |  |
| **Triple Condition Qualifying Comorbidity Sets** | | |
| Congestive Heart Failure | Heart Arrhythmias | Cerebrovascular Diseases |
| Congestive Heart Failure | Heart Arrhythmias | Coronary Artery Disease |
| Diabetes with Complications | Endocrine and Metabolic Disorders | Wheelchair/Beds |
| Diabetes with Complications | Heart Arrhythmias | Vascular Diseases |
| Diabetes with Complications | Vascular Diseases | Chronic Lung Diseases |
| Endocrine and Metabolic Disorders | Cerebrovascular Diseases | Complications Implants Graft |
| Endocrine and Metabolic Disorders | Congestive Heart Failure | Heart Arrhythmias |
| Endocrine and Metabolic Disorders | Chronic Lung Diseases | Complications Implants Graft |
| Endocrine and Metabolic Disorders | Complications Implants Graft | Coronary Artery Disease |
| Endocrine and Metabolic Disorders | Heart Arrhythmias | Complications Implants Graft |
| Endocrine and Metabolic Disorders | Heart Arrhythmias | Vascular Diseases |
| Endocrine and Metabolic Disorders | Vascular Diseases | Complications Implants Graft |
| Heart Arrhythmias | Complications Implants Graft | Coronary Artery Disease |
| Heart Arrhythmias | Vascular Diseases | Complications Implants Graft |
| Disorders of Immunity | Heart Arrhythmias | Vascular Diseases |
| Disorders of Immunity | Vascular Diseases | Chronic Lung Diseases |
| Vascular Diseases | Chronic Lung Diseases | Complications Implants Graft |
| Vascular Diseases | Chronic Lung Diseases | Coronary Artery Disease |
| Vascular Diseases | Complications Implants Graft | Coronary Artery Disease |

CKD = Chronic Kidney Disease

**Table S3.** Clinical Description of Comorbid Conditions for Input into the Multimorbid Patient Identifier App (MMApp).

Clinical labels of all comorbid conditions, included and excluded diagnoses, and associated Silber comorbid condition

| **Organ System** | **Silber Comorbid Condition** | **Clinical Comorbid Condition** | **Include** | **Exclude** |
| --- | --- | --- | --- | --- |
| Neurologic | Other Depression | Depression | Depressive Disorder | Bipolar Disorders, Schizoaffective Disorders |
|  | Substance Abuse with Complications | Substance Abuse, Dependence, Use or Overdose | Alcohol, Opioids, Cannabis, Sedatives/Hypnotics/Anxiolytics, Cocaine, Simulants, Hallucinogens, Inhalants, Psychoactive Substances, Narcotics | N/A |
|  | Cerebrovascular Diseases | Cerebrovascular Disease | Ischemic or hemorrhagic strokes including TIA (non-traumatic), Cerebrovascular Syndromes/Diseases, Spinal Cord Syndromes, Paralysis (full or partial) | Traumatic brain injury |
| Pulmonary | Home Oxygen Use | Home Oxygen Use | N/A | NA |
|  | Pneumonias | Pneumonia | Acute Pneumonia, Aspiration Pneumonitis, Lung/Mediastinal Abscess | N/A |
|  | Chronic Lung Diseases | Chronic Lung Diseases | COPD, Interstitial Lung Disease, Pneumonitis (excluding aspiration pneumonitis), Pulmonary Sequelae of Systemic Disease, Exposure/Inhalation-Induced Lung Diseases | Aspiration pneumonitis |
| Cardiac | Cardio and Respiratory Failure | Cardiac or Respiratory Failure | Ventricular Fibrillation/Flutter, Cardiac/Respiratory Arrest, Cardiogenic or Unspecified Shock (excluding septic shock) | Septic shock |
|  | Coronary Artery Disease | Coronary Artery Disease | N/A | Acute MI |
|  | Hypertension | Hypertension | N/A | N/A |
|  | Heart Arrhythmias | Cardiac Arrhythmias | Complete Heart Block | Ventricular Fibrillation/Flutter |
|  | Acute Myocardial Infarction | Acute Myocardial Infarction | STEMI, NSTEMI | N/A |
|  | Congestive Heart Failure | Heart Failure | Heart Failure, Cardiomyopathies, Pulmonary Embolism, Pulmonary Hypertension, Structural Diseases of the Pulmonary Vessels | N/A |
| Gastrointestinal | Liver Diseases | Liver Disease | Hepatitis (any cause), Cirrhosis, Acute or Chronic Hepatic Failure, Portal Hypertension, Esophageal Varices | N/A |
|  | Protein-Calorie Malnutrition | Protein-Calorie Malnutrition | Mild/Moderate/Severe Malnutrition, Cachexia | N/A |
| Genitourinary | Acute Renal Failure | Acute Kidney Injury | Any cause | N/A |
|  | CKD Stage 4-5 and Dialysis | CKD Stage 4-5 and Dialysis Dependence | Chronic Kidney Disease Stage 4-5 (GFR <30), End Stage Renal Disease/Dialysis Dependence | N/A |
| Endocrine | Diabetes with Complications | Diabetes with Complications | Diabetes with Complications, Diabetic Coma, Diabetic Nephropathy, Diabetic Eye Disease, Diabetic Neuropathy, Diabetic Vascular Disease, Diabetic Skin Disease, Diabetic Oral Disease | N/A |
|  | Endocrine and Metabolic Disorders | Endocrine or Metabolic Disorder | Metabolic/Genetic Disorders/Inborn Errors of Metabolism, Adrenal Disease, Hypothalamic/Pituitary Disease, Parathyroid Disease, MEN Syndromes, Thymus Disease, Porphyrias, Amyloidoses | N/A |
| Infectious Disease | Sepsis/Shock | Sepsis/Shock | Sepsis, Shock (excluding cardiogenic shock) | Cardiogenic Shock |
|  | Disorders of Immunity | Disorders of Immunity | Neutropenia/Pancytopenia, Immune Deficienceis (Innate or Acquired, excluding HIV/AIDS), Graft vs Host Disease, Hypoglobulinemia | HIV/AIDS |
|  | Opportunistic Infections | Opportunistic Infections | N/A | N/A |
| Vascular | Vascular Diseases | Vascular Diseases | Pulmonary Embolism, Peripheral Vascular Disease, Arterial Dissection or Aneurysm (excluding coronary and cerebrovascular), AV Fistula (excluding dialysis access), Phlebitis, Gangrene, Ischemic Vascular Disease (excluding coronary and cerebrovascular) | Coronary Aneurysm, Cerebrovascular Aneurysm, Dialysis Access, Coronary Ischemic Vascular Disease, Cerebrovascular Ischemic Vascular Disease |
| Skin | Pressure Ulcer, Skin | Pressure Ulcer, Skin | Pressure Ulcer of the Skin (any stage, any location) | N/A |
|  | Chronic Ulcer, Skin, Not Pressure | Chroinc Skin Ulcer (not pressure) | Diabetic Ulcer, Vascular Ulcer (arterial or venous), Unspecified Non-Pressure Skin Ulcer | N/A |
| Heme | Other Hematological | Hematologic Disease | Proliferative Hematologic Disease (excluding lymphoma/leukemia), Clotting Disorders, Platelet Disorders, Red Blood Cell Disorders (Excluding Sickle Cell and iron-deficiency anemia), Purpuras | Lymphoma/Leukemia, Sickle Cell and iron-deficinecy anemia |
| Other | Implants/Grafts with Complications | Complications from Implants/Grafts | Complication from any implanted catheter, graft, stent, prosthetic, or device | N/A |
|  | Amputation and Complications | Amputations +/- Complications | Traumatic or Non-traumatic amputation, Complications of amputations | N/A |
|  | Other Trauma | Trauma | Traumatic injury to the Spine or Extremity | N/A |
|  | Wheelchair/Beds | Wheelchair or Bed-Bound | N/A | N/A |

TIA = Transient Ischemic Attack; COPD = Chronic Obstructive Pulmonary Disease; MI = Myocardial Infarction; STEMI = ST Elevation Myocardial Infarction; NSTEMI = Non-ST Elevation Myocardial Infarction; CKD = Chronic Kidney Disease; GFR = Glomerular Filtration Rate; HIV = Human Immunodeficiency Virus; AIDS = Acquired Immunodeficiency Syndrome; AV = Arterio-Venous

**Section 2: MMApp Screenshots**

**Table S4.** MMApp Screen Progression for Non-Multimorbid Patient (Scenario D).

| **Screen Label** | **Screenshot** | **Note** |
| --- | --- | --- |
| Introduction Screen | 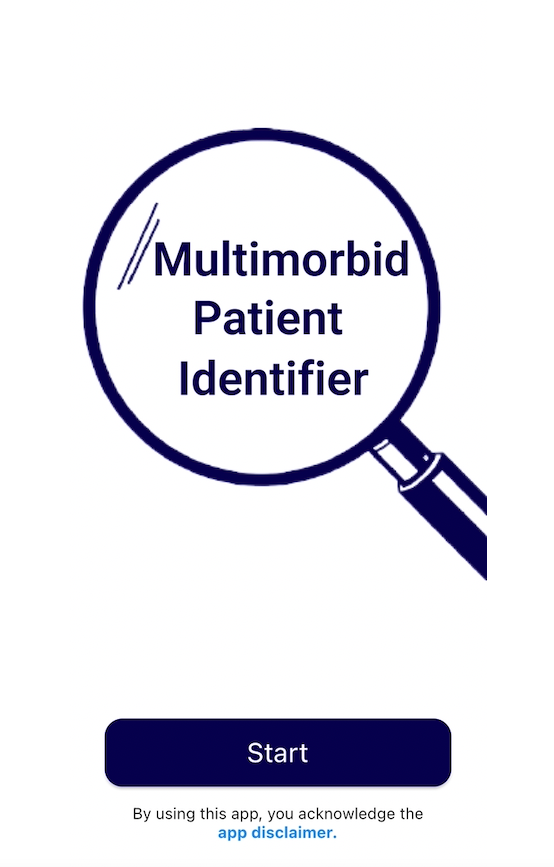 | Click “Start” to begin or can click on “app disclaimer” |
| ↓ | | |
| App Disclaimer | 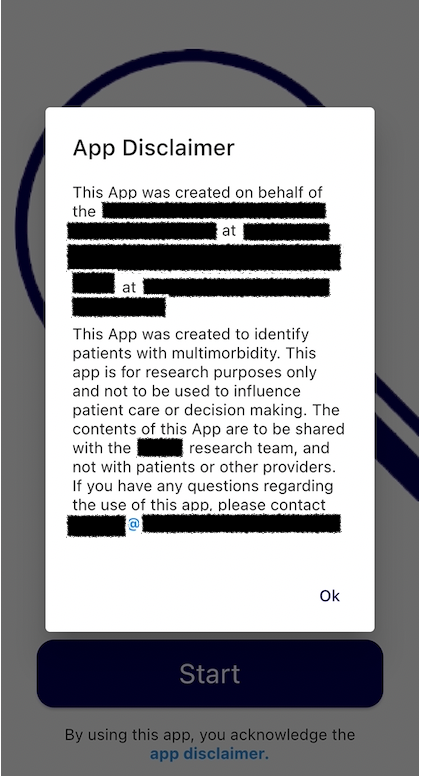 | Click “OK” to continue with app use or can click on **“*** @***.***”** to contact research team |
| ↓ | | |
| How to use this app informational page | 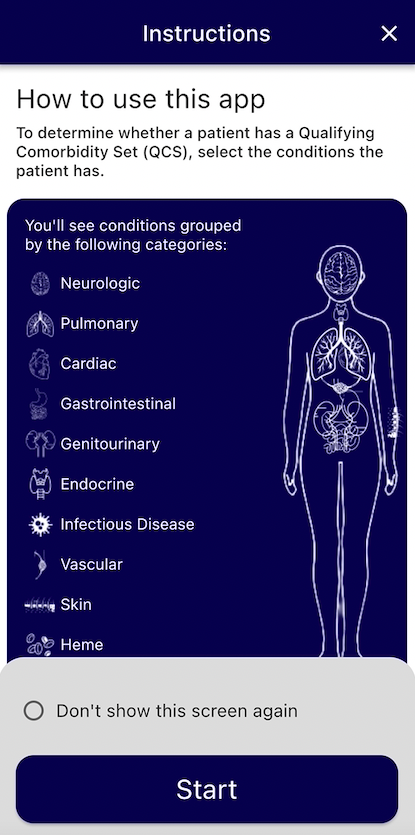 | Can click next to “Don’t show this screen again” if user already familiar with app |
| ↓ | | |
| Neurologic | 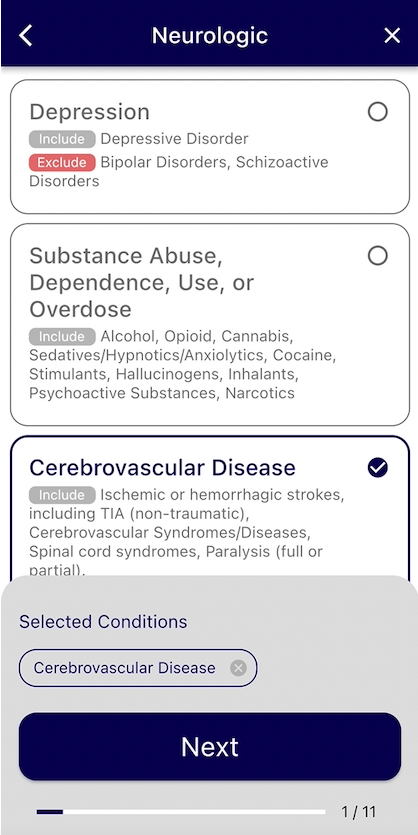 | Can click next to any condition if they are selected. For Scenario D, “Cerebrovascular Disease” is selected. Click “Next” to progress to next organ system. |
| ↓ | | |
| Pulmonary | 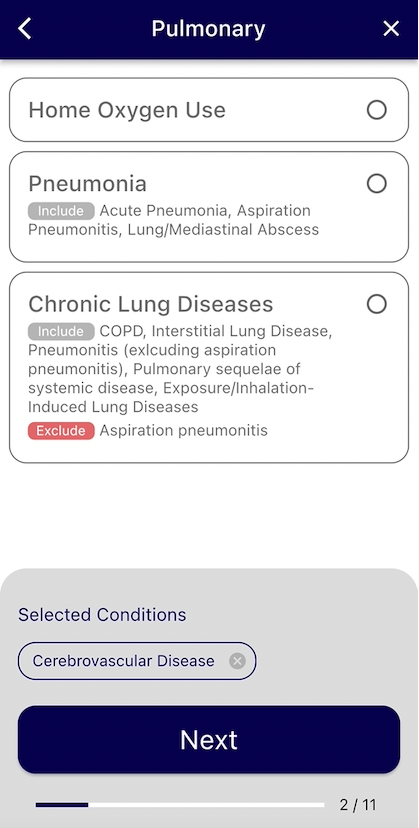 | No comorbidities are selected. Click “Next” to progress to next organ system. |
| ↓ | | |
| Cardiac | 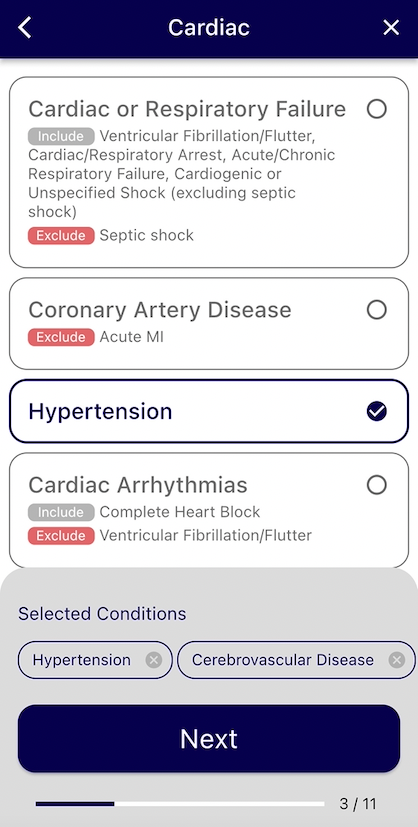 | “Hypertension” is selected. Click “Next” to progress to next organ system. |
| ↓ | | |
| Gastrointestinal | 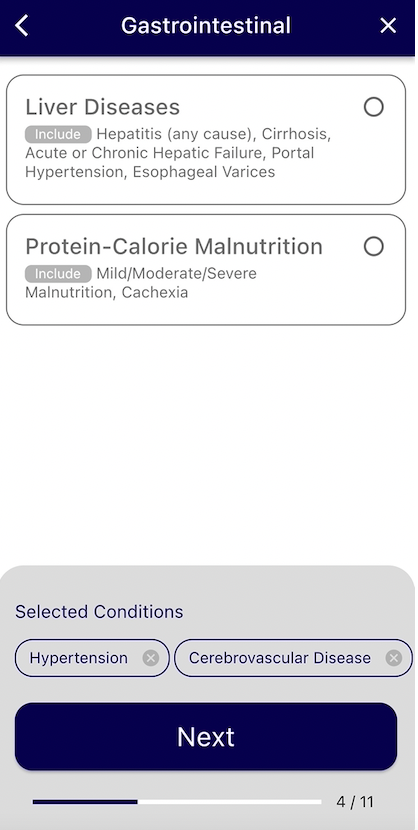 | No comorbidities are selected. Click “Next” to progress to next organ system. |
| ↓ | | |
| Genitourinary | 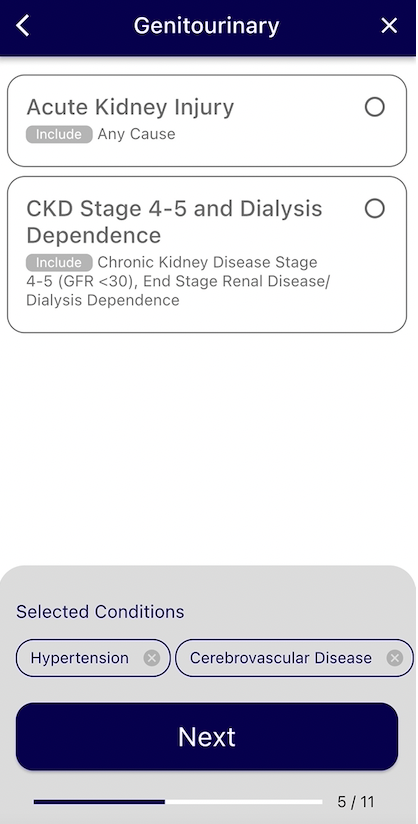 | No comorbidities are selected. Click “Next” to progress to next organ system. |
| ↓ | | |
| Endocrine | 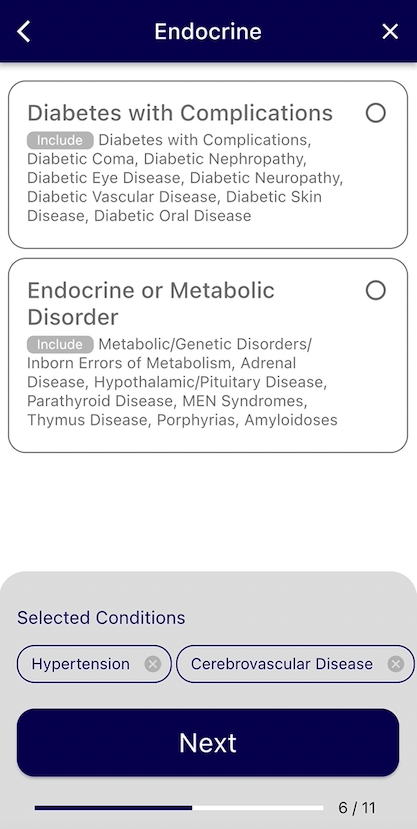 | No comorbidities are selected. Click “Next” to progress to next organ system. |
| ↓ | | |
| Infectious Disease | 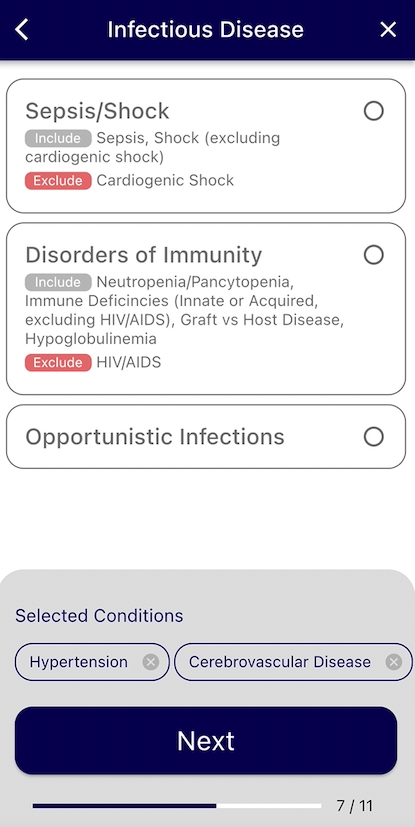 | No comorbidities are selected. Click “Next” to progress to next organ system. |
| ↓ | | |
| Vascular | 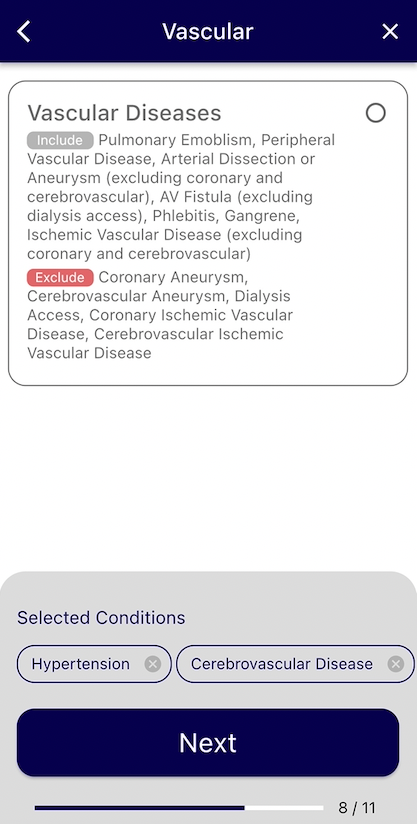 | No comorbidities are selected. Click “Next” to progress to next organ system. |
| ↓ | | |
| Skin | 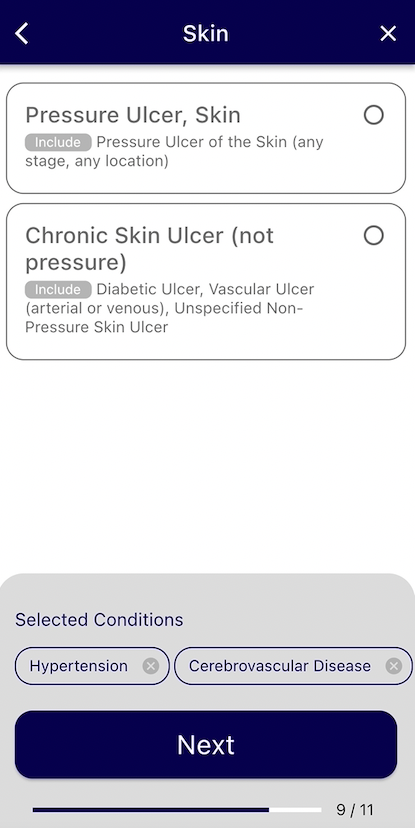 | No comorbidities are selected. Click “Next” to progress to next organ system. |
| ↓ | | |
| Heme | 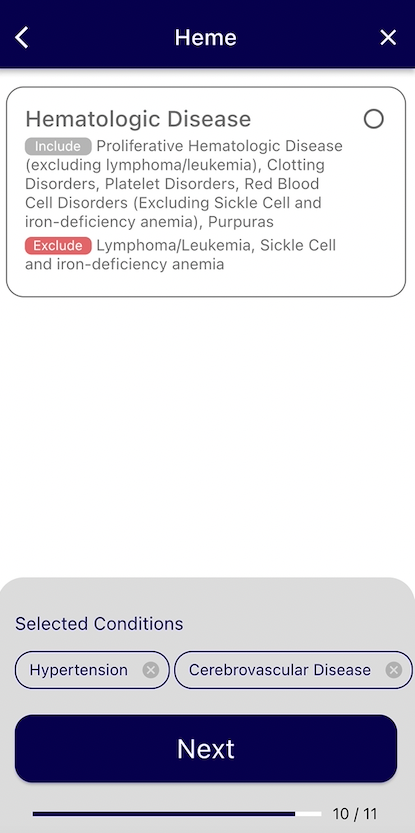 | No comorbidities are selected. Click “Next” to progress to next organ system. |
| ↓ | | |
| Other | 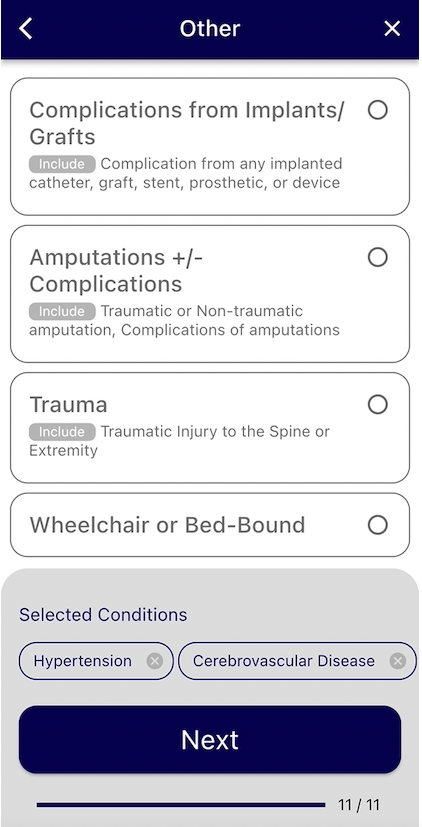 | No comorbidities are selected. Click “Next” to progress to next organ system. |
| ↓ | | |
| Results | 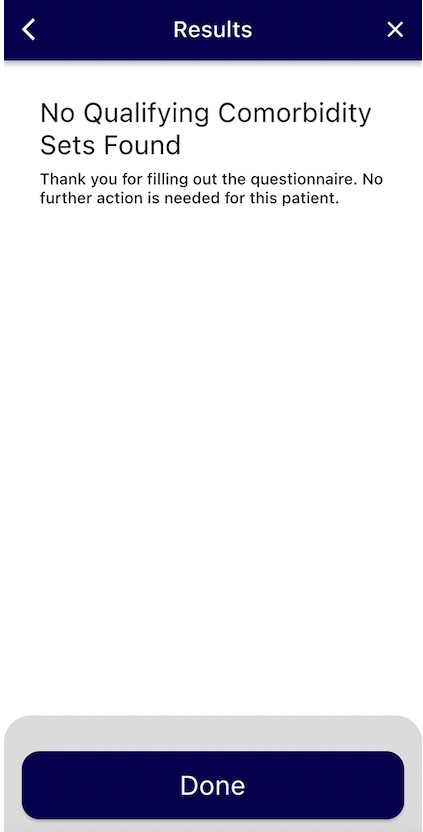 | Final page, no Qualifying Comorbidity Sets found so patient is not multimorbid. Click “Done” to begin again. |

* Note: Identifying Information of Research Team/Institution is hidden in this Supplement using black boxes.

**Table S5.** MMApp Screen Progression Example for Multimorbid Patient (Scenario E).

| Screen Label | Screenshot | Note |
| --- | --- | --- |
| Introduction Screen | 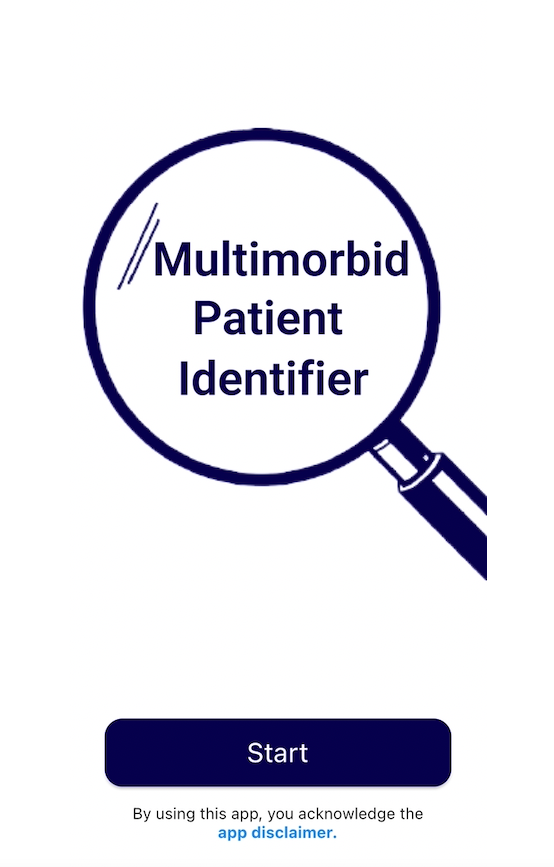 | Click “Start” to begin or can click on “app disclaimer” |
| ↓ | | |
| App Disclaimer | 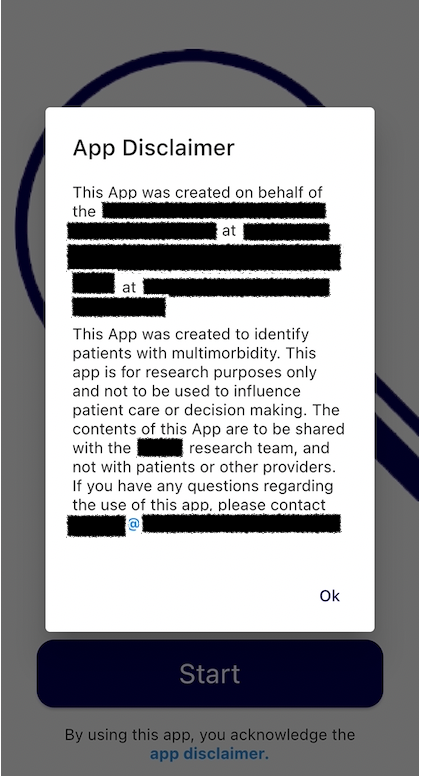 | Click “OK” to continue with app use or can click on **“***@***.***”** to contact research team |
| ↓ | | |
| How to use this app informational page | 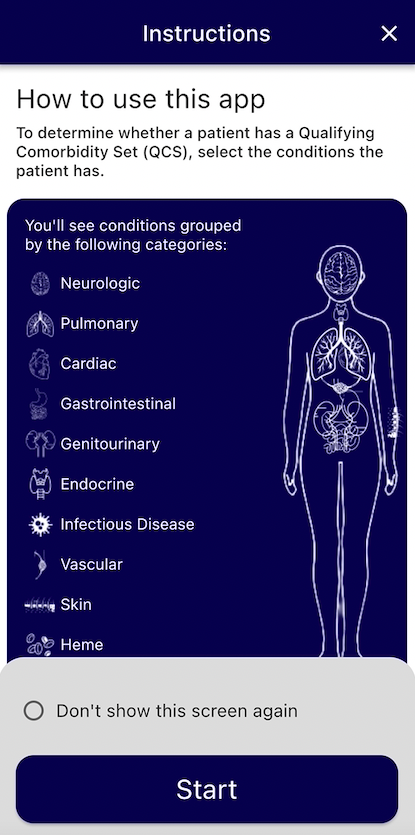 | Can click next to “Don’t show this screen again” if user already familiar with app |
| ↓ | | |
| Neurologic | 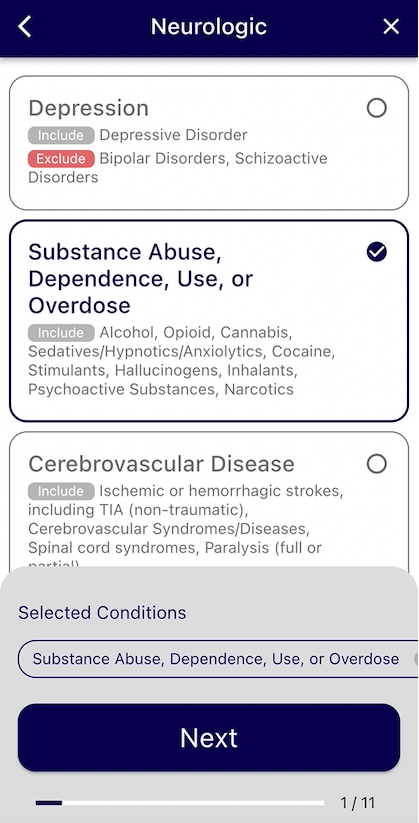 | Select “Substance Abuse, Dependence, Use, or Overdose”. Click “Next” to progress to next organ system |
| ↓ | | |
| Pulmonary | 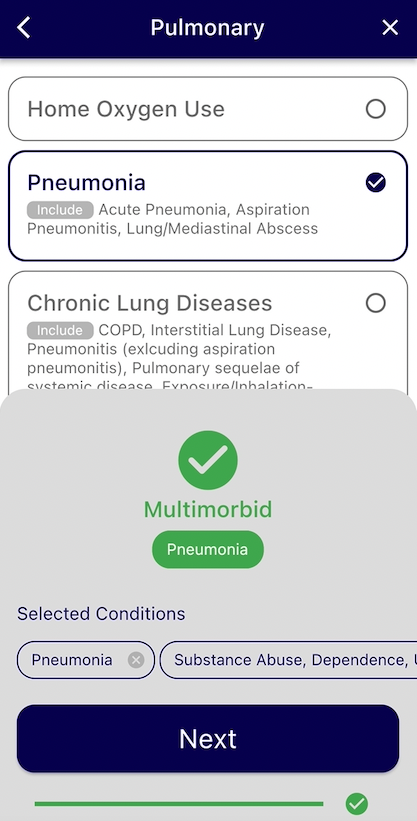 | Select “Pneumonia”. Patient identified as multimorbid. Click “next” to progress to next screen. |
| ↓ | | |
| Results | 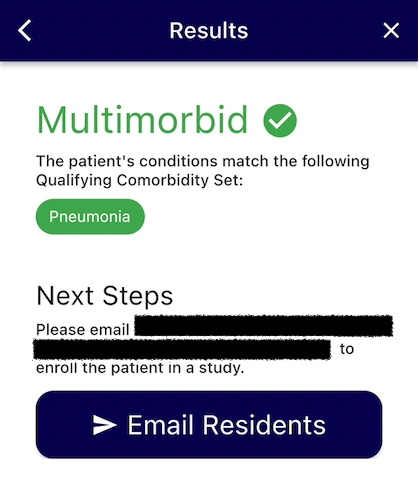 | Patient identified as multimorbid and satisfied Qualifying Comorbidity Set is displayed. Here it is a single condition of “Pneumonia” that satisfies the Qualifying Comorbidity Set. Click “Email Residents” to notify research team to potentially enroll patient in a study. |
| ↓ | | |
| Email | 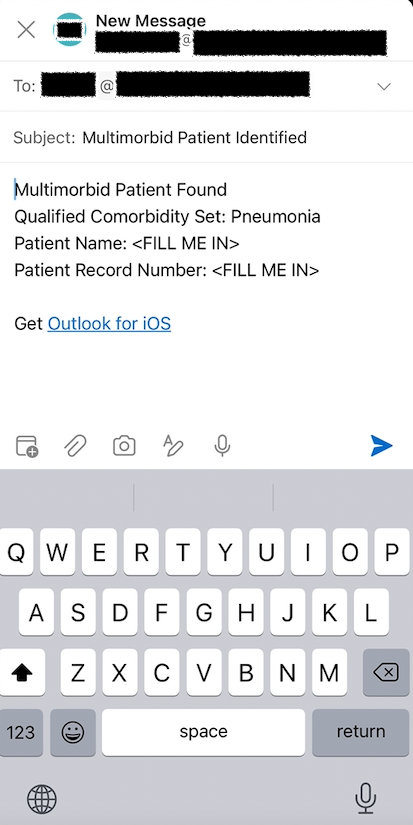 | An email is drafted to the research team including Qualifying Comorbidity Set. Can fill in Patient Name and Record Number as prompted. After sending email or hitting the “X” in the upper left-hand corner of the screen, will return to MMApp. |
| ↓ | | |
| Results | 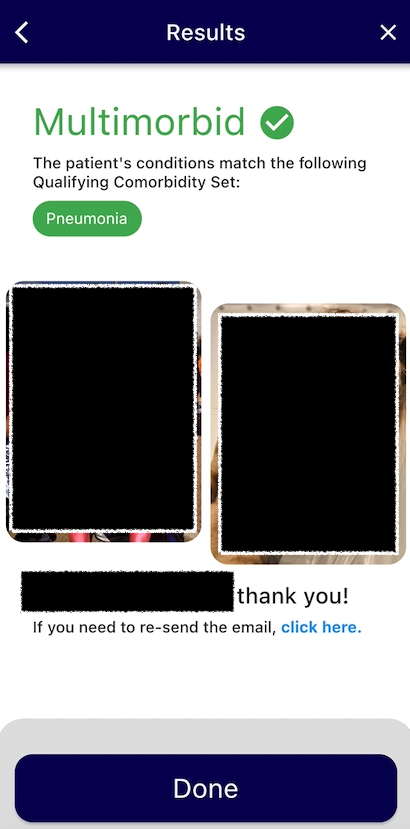 | Patient’s Qualifying Comorbidity Set again displayed, along with thank-you message from research Team and photos of research team. Option to re-send the same email as on the prior screen by clicking on “click here". Click “Done” to begin again. |

**Section 3: Hypothetical Patient Scenarios and Research Preamble**

**Information for users/Preamble** (read to participants)

Multimorbidity is the concept that certain combinations of comorbidities or patient characteristics make patients higher risk for complications than just having multiple comorbidities.

In the emergency surgery setting, multimorbid patients are at higher risk for complications than non-multimorbid patients, and we are currently working to quantify some of this risk in the research we do at the Center for Surgery and Health Economics. In order to investigate and operationalize the concept of multimorbidity, we need an efficient way to identify patients as multimorbid. As such, we have developed an app called the Multimorbid Patient Identifier to identify patients as multimorbid or not. The idea for the future is that after seeing a patient in the Emergency Department for an emergency surgery consult, you would input some of their history and current data to this app. The app would then let you know if the patient is multimorbid or not, and would enable you to email our research team with the patient’s information so that we could enroll them in studies to further investigate this concept. Before we can begin using this app for future investigation, we want to ensure that it is reasonable to use and easy to understand.

I would like you to work through the following five hypothetical scenarios. These are all scenarios of patients that you could be called to evaluate in the ED for an emergency general surgery condition. After reading through the case, please input the patient’s information into the Multimorbid Patient Identifier App. After that, please input that patient’s information into the ACS NSQIP risk calculator with the Procedure Code of 49000 (Exploratory Laparotomy). I will time you during these tasks and ask you a few questions afterwards. It should only take about 15-20 minutes of your time in total. Please do not ask me questions about how to use the app during the first scenario, but we will pause after the first scenario is completed so that you can ask any questions.

Your participation is completely voluntary and you are free to leave at any time. There is no compensation for your participation. The benefits of your participation are to our future research and the future practice of medicine. There are no perceived harms to you for participating.

**Table S6.** Abbreviation List for Hypothetical Patient Scenarios.

| **Abbreviation** | **Meaning** |
| --- | --- |
| @ | at |
| Alk Phos | Alkaline Phosphatase |
| ALT | alanine transaminase |
| AST | Aspartate Aminotransferase |
| b/l | bilateral |
| Bili | Bilirubin |
| BMI | Body Mass Index |
| BP | Blood Pressure (mmHg) |
| BUN | Blood Urea Nitrogen |
| Ca | Calcium |
| CBC | Complete Blood Count |
| CBD | Common bile duct |
| CC | Chief Complaint |
| CHF | Congestive Heart Failure |
| Cl | Chloride |
| CMP | Comprehensive Metabolic Panel |
| CO2 | Carbon Dioxide |
| Coags | Coagulation Labs |
| CPT | Common Procedural Terminology |
| Creat | Creatinine |
| CT | Computed Tomography |
| ED | Emergency Department |
| EtOH | Alcohol |
| Glu | Glucose |
| Hg | Hemoglobin |
| HLD | Hyperlipidemia |
| HPI | History of Present Index |
| HR | Heart Rate (beats per minute) |
| HTN | Hypertension |
| INR | International Normalized Ratio |
| IV | Intravenous |
| IVDU | Intravenous Drug Use |
| IVF | intravenous fluids |
| K | Potassium |
| Mg | Magnesium |
| MRN | Medical Record Number |
| Na | Sodium |
| NC | Nasal Cannula |
| NPO | nil per os (= nothing by mouth) |
| Phos | Phosphorus |
| Plt | Platelets |
| PMH | Past Medical History |
| PO | per os (= by mouth) |
| PSH | Past Surgical History |
| PT | Protime |
| PTT | Partial Thromboplastin Time |
| ROS | Review of Systems |
| RR | Respiratory Rate (breaths per minute) |
| subQ | subcutaneous |
| Temp | Temperature (Fahrenheit) |
| TIA | Transient Ischemic Attack |
| WBC | White Blood Cells |
| y/o | year old |

**Hypothetical Patient Scenario A**

**Scenario A:** Arnold Allen MRN 1234567

**CC**: Abdominal Pain

**HPI**

Mr. Allen is a 72 y/o M w PMH Afib (on Apixaban, last dose 2 days ago), HTN, and DM who presents to the ED with 2 days of nausea, vomiting, and abdominal pain. He reports that his last BM was 3 days ago. Last flatus was yesterday. He says that his abdomen feels “blown up”. He had an appendectomy 30 years ago, and denies any other surgical history. Last colonoscopy was 2 years ago and was normal. Has never had a bowel obstruction in the past.

**PMH**

- Atrial Fibrillation
- Hypertension
- Diabetes Mellitus (Type II)

**PSH**

- Appendectomy (open)
- R hip replacement (at 70 years old)

**Allergies**

- Patient has no known allergies

**Family History**

- Mother with a stroke (at 75, now deceased)
- Sister with breast cancer (diagnosed at 56)

**Social History**

- 1-2 beers per week
- Quit smoking 10 years ago (40 pack/year history)
- No illicit drug use
- Lives alone

**Medications**

Apixaban 5 mg twice per day, metformin 500 mg twice per day, amlodipine 5 mg every morning, HCTZ 25 mg daily

**ROS**

Negative aside from what is noted in HPI

**Physical Exam**

Temp 98.7 F HR 102 bpm BP 114/72 mmHg RR 12 respirations/minute O_2_Saturation 99% (Room Air) Height 5’10” Weight 183 lbs. BMI 27.19 kg/m^2^

NAD

HR normal

No respiratory distress

Abdomen distended, tympanic. Diffusely tender with mild rebound and guarding.

Mild b/l pitting edema

No rashes/bruises

**Labs**

CBC: WBC 12 Hg 13 Plt 212

CMP: Na 137 K 3.7 Cl 101 CO2 25 BUN 19 Creat 1.2 Glu 155 Ca 9 Mg 1.8 Phos 3 GFR 89

AST 28 ALT 31 Alk Phos 84 Total Bili 0.3 Direct Bili 0.1

Coags: PT 15.1 PTT 33.9 INR 1.3

Lactate 2

**Imaging**

Chest Xray: Left basilar atelectasis.

CT Abdomen/Pelvis with IV & PO Contrast: High-grade small bowel obstruction with transition point in the mid pelvis. No free air. No bowel wall pneumatosis. Surrounding mesenteric inflammation/edema. Recommend clinical correlation.

**Assessment/Plan:**

Mr. Allen is a 72 y/o M with PMH as above presenting with a high grade small bowel obstruction with abdominal tenderness including rebound/guarding.

- Plan for exploratory laparotomy (CPT 49000)
- NPO, IVF

Hold anticoagulation

**Hypothetical Patient Scenario B**

**Scenario B:** Beatrice Babington MRN 2234567

**CC**: Abdominal Pain

**HPI**

Ms. Babington is a 74 y/o F w PMH COPD, HTN, GERD, Chronic Back and knee pain, and depression/anxiety who presents to the ED with acute onset upper abdominal pain. She tends to have some baseline abdominal pain, but a few hours ago noted this severe upper abdominal pain. No fevers, but subjective chills. Some nausea, no vomiting. Passing gas, last BM this morning. She is brought in by her daughter (who lives with her) and the daughter reports that Ms. Babington has been a little sleepy and confused since the pain started.

**PMH**

- INFORMATION NOT PRESENT IN ELECTRONIC MEDICAL RECORD

**PSH**

- INFORMATION NOT PRESENT IN ELECTRONIC MEDICAL RECORD

**Allergies**

- INFORMATION NOT PRESENT IN ELECTRONIC MEDICAL RECORD

**Family History**

- INFORMATION NOT PRESENT IN ELECTRONIC MEDICAL RECORD

**Social History**

- No alcohol use
- Smokes ½ pack per day, 30 pack/year smoking history
- No illicit drug use
- Lives with daughter, uses a cane to ambulate

**Medications**

Albuterol inhaler 2 puffs as needed for wheezing, amlodipine 5 mg every morning, lansoprazole 30 mg daily before breakfast, tramadol 100 mg daily as needed for pain, Ativan 0.5 mg daily as needed for anxiety

**ROS**

Negative aside from what is noted in HPI

**Physical Exam**

Temp 101.3 F HR 118 bpm BP 102/64 mmHg RR 14 respirations/minute O_2_Saturation 100% (Room Air) Height 5’4” Weight 105 lbs. BMI 18 kg/m^2^

Uncomfortable, lethargic, thin

Tachycardic

Mild b/l wheezing, no rales/crackles/rhonchi

Abdomen mildly distended, tender with rebound/guarding in b/l upper quadrants

No rashes/bruises

**Labs**

CBC: WBC 14 Hg 12.8 Plt 149

CMP: Na 135 K 3.7 Cl 100 CO2 22 BUN 17 Creat 0.4 Glu 149 Ca 9 Mg 1.2 Phos 3

AST 28 ALT 31 Alk Phos 84 Total Bili 0.3 Direct Bili 0.1

Coags: PT 12.3 PTT 33.9 INR 0.8

Lactate 3.4

**Imaging**

Chest Xray: b/l mild hyperinflation

CT Abdomen/Pelvis with IV & PO Contrast: inflammation surrounding first part of duodenum with adjacent leakage of oral contrast. Moderate free air, concerning for perforated peptic ulcer. Correlate clinically.

**Assessment/Plan:**

Ms. Babington is a 74 y/o F with PMH as above presenting with concern for perforated peptic ulcer.

- Plan for exploratory laparotomy (CPT 49000)
- NPO, IVF

**Hypothetical Patient Scenario C**

**Scenario C: Carl Crest MRN 3234567**

**CC:** Abdominal Pain

**HPI**

Mr. Crest is a 66 y/o M w PMH CHF, HTN, HLD and Peripheral arterial disease who presents to the ED with acute onset lower abdominal pain. He reports some mild lower abdominal tenderness beginning a few days ago, but that this morning it got acutely worse. He reports fevers and chills. He reports nausea for a few days but no vomiting. Also has had diarrhea for a few days. His last colonoscopy was 7 years ago and was normal.

**PMH**

- Congestive heart failure
- Hypertension
- Peripheral arterial disease
- Hyperlipidemia

**PSH**

- Laparoscopic appendectomy
- Umbilical hernia repair

**Allergies**

- Penicillin

**Family History**

- Father with diabetes and stroke at age 76

**Social History**

- No alcohol use
- No tobacco use
- No illicit drug use
- Lives with wife

**Medications**

Lasix 10 mg daily, lisinopril 5 mg daily, amlodipine 10 mg daily, metoprolol 50 mg twice daily, atorvastatin 20 mg daily

ROS

Negative aside from what is noted in HPI

**Physical Exam**

Temp 99 F HR 99 bpm BP 105/72 mmHg RR 12 respirations/minute O_2_Saturation 100% (Room Air) Height 5’11” Weight 185 lbs. BMI 26 kg/m^2^

Uncomfortable, moderate distress

HR normal

Mild b/l crackles @ lung bases

Tender in b/lw lower quadrants with rebound and guarding

b/l lower extremity 2+ pitting edema

**Labs**

CBC: WBC 15 Hg 12.8 Plt 149

CMP: Na 135 K 3.7 Cl 100 CO2 22 BUN 50 Creat 2.4 Glu 149 Ca 9 Mg 1.2 Phos 3

AST 28 ALT 31 Alk Phos 84 Total Bili 0.3 Direct Bili 0.1

Coags: PT 12.3 PTT 33.9 INR 0.8

Lactate 3.4

**Imaging**

CT Abdomen/Pelvis with IV & PO Contrast: Inflammation and stranding around sigmoid colon with leakage of contrast and free air concerning for perforated diverticulitis.

**Assessment/Plan:**

Mr. Crest is a 66 y/o M with PMH as above presenting with concern for perforated diverticulitis

- Plan for exploratory laparotomy (CPT 49000)
- NPO, IVF

**Hypothetical Scenario D**

**Scenario D:** Dorreen Davidson MRN 4234567

**CC**: Abdominal Pain

**HPI**

Ms. Davidson is a 71 y/o F w PMH TIA, HTN, and HLD who presents to the ED with upper abdominal pain that began yesterday after dinner. She reports being in her usual state of health, but yesterday after dinner developed upper abdominal pain and nausea and had one episode of non-bloody/nonbilious emesis. She reports the pain as crampy in nature. It has not gotten any better, and she was unable to sleep last night because of the pain.

**PMH**

- Transient Ischemic Attack
- Hypertension
- Hyperlipidemia

**PSH**

- None

**Allergies**

- No known drug allergies

**Family History**

- Father with diabetes and stroke at age 76

**Social History**

- No alcohol use
- Quit smoking 5 years ago (35 pack/year smoking history)
- No illicit drug use
- Lives with wife

**Medications**

Amlodipine 10 mg daily, Metoprolol 50 mg twice daily, Atorvastatin 20 mg daily, Insulin Glargine 10 units subQ every night, Insulin Aspart 4 units subQ with meals

**ROS**

Negative aside from what is noted in HPI

**Physical Exam**

Temp 99 F HR 99 bpm BP 112/72 mmHg RR 12 respirations/minute O_2_Saturation 100% (Room Air) Height 5’4” Weight 165 lbs. BMI 28 kg/m^2^

No acute distress

HR normal

No respiratory distress

Tenderness in right upper quadrant with + Murphy’s sign

No edema, no rashes/skin lesions

**Labs**

CBC: WBC 12 Hg 12.8 Plt 149

CMP: Na 135 K 3.7 Cl 100 CO2 22 BUN 18 Creat 0.9 Glu 172 Ca 9 Mg 1.2 Phos 3

AST 47 ALT 52 Alk Phos 102 Total Bili 0.9 Direct Bili 0.6

Coags: PT 12.3 PTT 33.9 INR 0.8

Lactate 1.4

**Imaging**

Chest Xray: normal

Abdominal Ultrasound: Gallbladder wall thickening of 3.4 mm with distended gallbladder and mild pericholecystic fluid. Positive sonographic Murphy’s Sign. CBD 6 mm in diameter.

**Assessment/Plan:**

Ms. Davidson is a 71 y/o M with PMH as above presenting with concern for acute cholecystitis

- Plan for exploratory laparotomy (CPT 49000)
- NPO, IVF
- IV ceftriaxone/metronidazole

**Hypothetical Scenario E**

**Scenario E:** Ernest Elmers MRN 5234567

**CC**: Abdominal Pain

**HPI**

Mr. Elmers is a 82 y/o M w EtOH abuse, HTN, HLD, and mild cirrhosis who presents to the ED with diffuse abdominal pain, nausea and vomiting for the past 4 days. His last bowel movement was 5 days ago. He has not passed flatus in 2 days. He has had 4 prior bowel obstructions, no prior operations.

**PMH**

- EtOH Abuse
- Hypertension
- Hyperlipidemia
- Cirrhosis

**PSH**

- Open cholecystectomy (40 years ago)

**Allergies**

- No known drug allergies

**Family History**

- Father with diabetes and stroke at age 76

**Social History**

- 4-6 beers per day
- No tobacco use
- Prior IVDU with hepatis
- Lives alone

**Medications**

Metoprolol 50 mg twice daily, Atorvastatin 20 mg daily

ROS

Negative aside from what is noted in HPI

**Physical Exam**

Temp 102 F HR 114 bpm BP 100/60 mmHg RR 12 respirations/minute O_2_Saturation 94% (4L NC) Height 5’7” Weight 155 lbs. BMI 25 kg/m^2^

Uncomfortable, moderate distress

Tachycardic

Notable respiratory distress

Diffuse tenderness with rebound/guarding. Distended/tympanic abdomen. Pain with jostling of the patient’s stretcher.

B/l pitting edema

**Labs**

CBC: WBC 12 Hg 13 Plt 212

CMP: Na 133 K 3.7 Cl 101 CO2 25 BUN 19 Creat 1.2 Glu 155 Ca 9 Mg 1.8 Phos 3 GFR 89

AST 28 ALT 31 Alk Phos 84 Total Bili 0.4 Direct Bili 1

Coags: PT 15.1 PTT 33.9 INR 1.3

Lactate 3.4

**Imaging**

Chest Xray: Right lower lobe infiltrate, concerning for aspiration pneumonia

CT abdomen/pelvis with IV and PO contrast: High grade small bowel obstruction with greatest diameter of small bowel to 5.5 cm, moderate free intraabdominal air.

**Assessment/Plan:**

Mr. Elmers is a 71 y/o M with PMH as above presenting with concern for perforated small bowel obstruction

- Plan for exploratory laparotomy (CPT 49000)
- NPO, IV
- IV vancomycin/piperacillin-tazobactam

**Comorbidities Present for Each Scenario**

- Scenario A = Non-Multimorbid Patient
  - Conditions present
    - “Hypertension”
    - “Cardiac Arrhythmias”
- Scenario B = Multimorbid Patient
  - Conditions Present
    - “Depression”
    - “Chronic Lung Disease”
    - “Hypertension”
    - “Protein-Calorie Malnutrition
      - Note: MMApp would terminate here if all are chosen
    - “Sepsis/Shock”
- Scenario C = Multimorbid Patient
  - Conditions Present
    - “Hypertension”
    - “Heart Failure”
    - Acute Kidney Injury”
      - Note: MMApp would terminate here if all are chosen
    - Vascular Diseases
- Scenario D = Non-Multimorbid Patient
  - Conditions Present
    - “Cerebrovascular Disease”
    - “Hypertension”
- Scenario E
  - Conditions Present
    - “Substance Abuse”
    - “Pneumonia”
      - Note: MMApp would terminate here if all are chosen
    - “Hypertension”
    - “Liver Disease”

**Section 4: MMApp Survey**

| I often use a risk calculator like NSQIP when evaluating patients | \| Strongly disagree \| Disagree \| Neutral \| Agree \| Strongly Agree \| \| --- \| --- \| --- \| --- \| --- \| \|  \|  \|  \|  \|  \| |
| --- | --- | --- | --- | --- | --- | --- | --- | --- | --- | --- | --- |
| What did you think of the Multimorbid Patient Identifier App? | |
| - It was easy to use | \| Strongly disagree \| Disagree \| Neutral \| Agree \| Strongly Agree \| \| --- \| --- \| --- \| --- \| --- \| \|  \|  \|  \|  \|  \| |
| - It was good to use | \| Strongly disagree \| Disagree \| Neutral \| Agree \| Strongly Agree \| \| --- \| --- \| --- \| --- \| --- \| \|  \|  \|  \|  \|  \| |
| - The time spent using it has been acceptable | \| Strongly disagree \| Disagree \| Neutral \| Agree \| Strongly Agree \| \| --- \| --- \| --- \| --- \| --- \| \|  \|  \|  \|  \|  \| |
| - The introduction of how to use it was sufficient | \| Strongly disagree \| Disagree \| Neutral \| Agree \| Strongly Agree \| \| --- \| --- \| --- \| --- \| --- \| \|  \|  \|  \|  \|  \| |
| - I needed more training on how to use the app | \| Strongly disagree \| Disagree \| Neutral \| Agree \| Strongly Agree \| \| --- \| --- \| --- \| --- \| --- \| \|  \|  \|  \|  \|  \| |
| - I would recommend it to others | \| Strongly disagree \| Disagree \| Neutral \| Agree \| Strongly Agree \| \| --- \| --- \| --- \| --- \| --- \| \|  \|  \|  \|  \|  \| |
| - It changes the way I would think about my patient | \| Strongly disagree \| Disagree \| Neutral \| Agree \| Strongly Agree \| \| --- \| --- \| --- \| --- \| --- \| \|  \|  \|  \|  \|  \| |
| - It was too time consuming | \| Strongly disagree \| Disagree \| Neutral \| Agree \| Strongly Agree \| \| --- \| --- \| --- \| --- \| --- \| \|  \|  \|  \|  \|  \| |
| - It was boring to use | \| Strongly disagree \| Disagree \| Neutral \| Agree \| Strongly Agree \| \| --- \| --- \| --- \| --- \| --- \| \|  \|  \|  \|  \|  \| |
| - I think the app would be distracting from my work/patient care | \| Strongly disagree \| Disagree \| Neutral \| Agree \| Strongly Agree \| \| --- \| --- \| --- \| --- \| --- \| \|  \|  \|  \|  \|  \| |
| - The app was intuitive to use | \| Strongly disagree \| Disagree \| Neutral \| Agree \| Strongly Agree \| \| --- \| --- \| --- \| --- \| --- \| \|  \|  \|  \|  \|  \| |
| - A risk calculator like the app would be helpful | \| Strongly disagree \| Disagree \| Neutral \| Agree \| Strongly Agree \| \| --- \| --- \| --- \| --- \| --- \| \|  \|  \|  \|  \|  \| |
| - I would be likely to use the app | \| Strongly disagree \| Disagree \| Neutral \| Agree \| Strongly Agree \| \| --- \| --- \| --- \| --- \| --- \| \|  \|  \|  \|  \|  \| |
| - I would be likely to use the app in my practice if it provided me with risk information for my patient | \| Strongly disagree \| Disagree \| Neutral \| Agree \| Strongly Agree \| \| --- \| --- \| --- \| --- \| --- \| \|  \|  \|  \|  \|  \| |
| What features of the app do you find easy to use? |  |
| What features of the app do you find confusing? |  |
| Do you have any suggestions for improving the app? |  |

Survey adapted from Melin et al. [31] and Mummah et al. [30]

**Section 6: Sensitivity and Specificity of Identifying Multimorbidity with MMApp**

**Table S7.** Multimorbidity Identification with MMApp.

|  | Identified as Multimorbid with MMApp | Identified as Non-Multimorbid with MMApp | Total |
| --- | --- | --- | --- |
| Multimorbid Patient | 29 trials (True Positive) | 1 trial (False Negative) | 30 trials |
| Non-Multimorbid Patient | 1 trial (False Positive) | 19 trials (True Negative) | 20 trials |
| Total | 30 trials | 20 trials | 50 trials |

MMApp = Multimorbid Patient Identifier App

Sensitivity = (True Positive)/ (True Positive + False Positive) = 29/30 = 96.7%

Specificity = (True Negative)/ (True Negative + False Negative) = 19/20 = 95%
